# Co-expression Gene Networks and Machine-learning Algorithms Unveil a Core Genetic Toolkit for Reproductive Division of Labour in Rudimentary Insect Societies

**DOI:** 10.1093/gbe/evac174

**Published:** 2022-12-17

**Authors:** Emeline Favreau, Katherine S Geist, Christopher D R Wyatt, Amy L Toth, Seirian Sumner, Sandra M Rehan

**Affiliations:** Department of Genetics, Environment, Evolution, University College London, London WC1E 6BT, United Kingdom; Department of Ecology, Evolution, and Organismal Biology, Iowa State University, Ames, Iowa 50011; Department of Genetics, Environment, Evolution, University College London, London WC1E 6BT, United Kingdom; Department of Ecology, Evolution, and Organismal Biology, Iowa State University, Ames, Iowa 50011; Department of Genetics, Environment, Evolution, University College London, London WC1E 6BT, United Kingdom; Department of Biology, York University, Toronto, ON M3J 1P3, Canada

**Keywords:** wasps, bees, SVM, RNAseq, castes, sociality, social insects, simple societies

## Abstract

The evolution of eusociality requires that individuals forgo some or all their own reproduction to assist the reproduction of others in their group, such as a primary egg-laying queen. A major open question is how genes and genetic pathways sculpt the evolution of eusociality, especially in rudimentary forms of sociality—those with smaller cooperative nests when compared with species such as honeybees that possess large societies. We lack comprehensive comparative studies examining shared patterns and processes across multiple social lineages. Here we examine the mechanisms of molecular convergence across two lineages of bees and wasps exhibiting such rudimentary societies. These societies consist of few individuals and their life histories range from facultative to obligately social. Using six species across four independent origins of sociality, we conduct a comparative meta-analysis of publicly available transcriptomes. Standard methods detected little similarity in patterns of differential gene expression in brain transcriptomes among reproductive and non-reproductive individuals across species. By contrast, both supervised machine learning and consensus co-expression network approaches uncovered sets of genes with conserved expression patterns among reproductive and non-reproductive phenotypes across species. These sets overlap substantially, and may comprise a shared genetic “toolkit” for sociality across the distantly related taxa of bees and wasps and independently evolved lineages of sociality. We also found many lineage-specific genes and co-expression modules associated with social phenotypes and possible signatures of shared life-history traits. These results reveal how taxon-specific molecular mechanisms complement a core toolkit of molecular processes in sculpting traits related to the evolution of eusociality.

**Significance**

Insects societies range from simplicity—as two totipotent females caring for few offspring, to complexity—as a committed honeybee queen supported by thousands of partially sterile workers. Understanding how social behaviors are regulated in rudimentary forms of sociality is fundamental for uncovering the foundations of social evolution. Yet, we lack a standardized test of this across independent social lineages at the molecular level. Using a combination of machine learning and co-expression analyses applied in a standardized manner across multiple datasets, we uncover a core genetic toolkit for social behavior that is shared across a range of lineages. Our continuing quest to understand the emergence of rudimentary sociality must capture and account for the contributions of evolutionary history and life-history to the building blocks of social life.

## Introduction

Sociality fascinated and flummoxed Darwin (Darwin 1859; Ratnieks et al. 2011)—how can evolution produce individuals who sacrifice reproduction to promote the reproduction of others? Inclusive fitness theory is widely invoked to delineate how altruistic, non-reproductive individuals can evolve by passing on their genes via relatives who are dedicated reproductives (Hamilton 1964; Bourke and Franks 1995; West et al. 2015). In recent years, there has been a shift in focus to understand the proximate machinery by which the genome of a single species can give rise to the alternative social phenotypes—reproductives (queens) and non-reproductives (workers) (Strassmann et al. 1989; Bourke and Franks 1995; Crozier et al. 1996; Rittschof and Robinson 2016; Toth and Rehan 2017; Kapheim 2018). It is remarkable that these same phenotypic solutions to social living have evolved at least eight times independently within the Hymenoptera (bees, wasps, and ants) (Hughes et al. 2008). Studies on the molecular basis of social phenotypes in these insects have shown how over evolutionary time genotypes have been co-opted, adapted, evolved, and/or converged to produce alternative phenotypic expressions of shared genomes (Evans and Wheeler 2001; Hunt et al. 2013; Simola et al. 2013; Berens et al. 2015; Toth and Rehan 2017; Weitekamp et al. 2017; Warner et al. 2019). Despite the multitude of data, our ability to search for common or contrasting patterns of molecular machinery across different datasets is limited by incompatibilities between datasets, arising from the rapidly changing methods in molecular biology; moreover, the influence of ecology, life-history, lineage, and level of social complexity (Wilson 1971) have been largely overlooked.

Sociality pervades the tree of life in all shapes and sizes, from the emblematic social insects to the enigmatic slime molds. In many cases, the social group is temporary and ephemeral: group members retain autonomy and have the power to exercise different gene-propagation strategies as non-reproductives or reproductives and they can change what they do over time, depending on intrinsic or extrinsic factors. These traits describe rudimentary societies, and can be found amongst the *Dictyostelium* slime molds, *Polistes* paper wasps and some *Ceratina* small carpenter bees. Some may have the option to choose between the social option and living alone; such as mongooses, halictid bees, and stenogastrine wasps. Other types of societies, less rudimentary, have become groups of specialists, with each unit component becoming committed to a specific reproductive or non-reproductive role, and being mutually dependent on each other; so complex are these societies that they constitute a major transition to a new level of individuality in their own right (Szathmáry and Smith 1995). These complex societies are epitomized in Hymenoptera by honeybees, vespine wasps, most ants.

A comprehensive understanding of *how* altruists and their beneficiaries can arise from the same genome remains elusive, especially given the 200 Myr of Hymenoptera evolution into a large diversity of biological complexities, enhanced by a proportioned research effort producing far more datasets from complex societies than simpler societies (Branstetter et al. 2018). Genomic and transcriptomic analyses of reproductive and non-reproductive phenotypes (hereafter named social phenotypes) in Hymenoptera provide emerging evidence for two overarching, but contrasting, patterns on the evolutionary nature of the machinery that makes social phenotypes. The most prominent pattern is that evolution appears to often co-opt the same aspects of the genome to generate social phenotypes, suggesting an important role for a so-called “genetic toolkit” for sociality (Toth and Robinson 2007). The toolkits include specific genes that are shared across species and differentially expressed between social phenotypes; for example, genes related to core metabolic and reproductive processes (e.g., the egg-yolk protein Vitellogenin (Amsalem et al. 2014; Morandin et al. 2019)), possible “master” regulatory genes (e.g., zinc finger transcription factor family [Rehan et al. 2018]), and genes related to neural and sensory processing (e.g., *neuroparsin-A-like* [Qiu et al. 2018]). Signs of a toolkit for sociality are also evident at the functional level, with numerous studies uncovering shared gene pathways (Berens et al. 2015), networks (Patalano et al. 2015; Morandin et al. 2016), and molecular and cellular processes at the mRNA level between queen and worker social phenotypes (Wyatt et al. 2020; Shell and Rehan 2022). Genes upregulated in reproductives may relate to epigenetic modifications (Shell and Rehan 2019) or conserved functions such as transcription or biosynthetic processes associated with basic functions (Taylor et al. 2021). Along-side genetic toolkits for sociality are signs that evolution sometimes uses taxon-specific mechanisms that may have evolved de novo to produce alternative phenotypes (Sumner 2014). For example, genes that are evolutionarily younger (or taxonomically restricted to specific taxa), have been found to be frequently associated with caste-related gene expression (Johnson and Tsutsui 2011; Ferreira et al. 2013; Feldmeyer et al. 2014; Berens et al. 2015; Rehan and Toth 2015; Shell et al. 2021). Comparative sociogenomics across independent lineages to date have compared across levels of social complexity, for example rudimentary *Polistes* societies versus highly derived *Apis mellifera* societies (Patalano et al. 2015) or within a single origin of sociality (Wyatt et al. 2020; Shell et al. 2021). However, we have yet to understand the extent to which there are shared patterns associated with social phenotypes in rudimentary societies of bees and wasps, using comparable methods of analyses. This information is critical to develop a comprehensive mechanistic scenario, on a molecular level, of how eusociality can evolve.

Here, we conducted a meta-analysis of existing transcriptomic data for social phenotypes from six species of bees and wasps, representing independent origins of rudimentary forms of sociality, thus providing a much-needed standardized cross-species assessment of the molecular basis of alternative social phenotypes (reproductives and non-reproductives). We focus our efforts on species exhibiting rudimentary societies—including both facultatively and obligately social species; thus, they vary in level of social complexity, but they all share the trait of alternative female social phenotypes (or rudimentary “castes”) which exploit different reproductive roles. These types of societies are likely to be the most informative for understanding how altruism might emerge when social groups first form from a solitary ancestor; although these species do not necessarily represent the first societies to evolve, their phylogenetic placement and life history traits make them a useful proxy among extant species (Rehan and Toth 2015). Comparisons of brain transcriptomic data for these six species, therefore, allow us to test the extent to which a conserved genetic toolkit may be related to the evolution of altruism in bees and wasps (Hypothesis 1: reproductive phenotype). Support for the toolkit hypothesis predicts significant similarity in expression across all six species at the gene and/or gene module level, specifically for some of deeply conserved genes and pathways related to reproduction and regulation of core forms of behavior (Amsalem et al. 2014; Qiu et al. 2018; Rehan et al. 2018; Morandin et al. 2019).

**Fig. 1.**
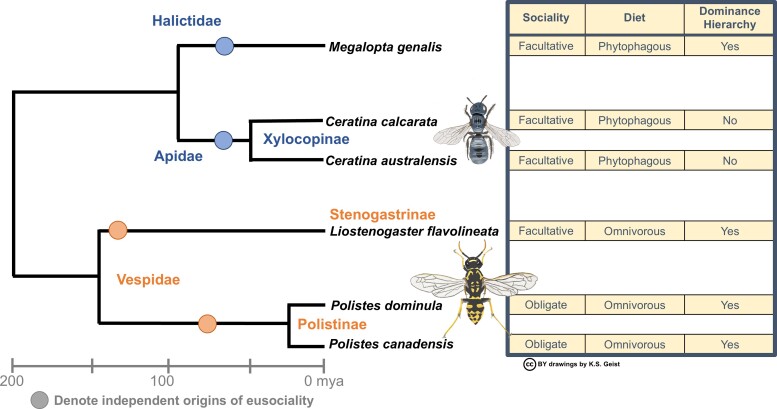
Phylogeny with four independent origins of sociality (circles) for bees (Halictidae & Apidae) and wasps (Vespidae: Polistinae & Stenogastrinae) representing a range of social and ecological phenotypes (traits in table on the right). Additional behavioral and phenotypic information on each species can be found in supplementary Tables S1 and S2, Supplementary Material online.

Our six species also represent four independent origins of sociality—two in the bees (∼100 Ma between *Megalopta genalis* and *Ceratina spp.* [Peters et al. 2017]) and two in the wasps (∼166 Ma between *Liostenogaster flavolineata* and *Polistes spp*. [Huang et al. 2019]) (fig. 1). We include congeners in each group; these permit testing of how social lineage and phylogenetic relatedness influences the degree to which molecular processes are shared. Given the possible importance of taxonomically restricted genes in caste determination, we predict that patterns of gene expression in more closely related species (e.g., congeners; family, or social lineage) may more closely mirror each other than those with more distant evolutionary relationships (Hypothesis 2: phylogenetic clade). Accordingly, these six species offer the opportunity for a rigorous test of the extent to which there is a shared set of proximate molecular processes regulating altruistic behaviors, taking account of the level of phenotypic specialism and commitment shown by altruists, and account of lineage, ecology, and life history (fig. 1, supplementary Table S2, Supplementary Material online).

## Results

Using publicly available datasets, we compared head or brain transcriptomes of reproductives and non-reproductive phenotypes from each of the following six species: the halictid bee *M. genalis* (Jones et al. 2017), the xylocopine bees *Ceratina australensis* (Rehan et al. 2018), and *Ceratina calcarata* (Shell and Rehan 2019), the stenogastrine wasp *L. flavolineata* (Taylor et al. 2022), and the polistine wasps *Polistes canadensis* (Patalano et al. 2015) and *Polistes dominula* (Taylor et al. 2021) (sampling details in supplementary Table S4, Supplementary Material online). The quality and completeness of the mapped RNAseq reads are comparable across the six species, despite variation in the level of replication across the species (*n* = 6–24 RNAseq samples per species, supplementary Table S5, Supplementary Material online). We identified 3,718 nearly single-copy orthologs across the two clades (supplementary Table S6, Supplementary Material online). Within clades we identified 5,787 nearly single-copy orthologs across the three bee species, and 6,983 across the three wasps. All subsequent analyses focused on orthologous gene sets, unless named otherwise as species-specific.

### Overall Patterns of Gene Expression Cluster by Phylogeny Rather Than by Social Phenotype

To first examine whether overall brain gene expression clusters more by phylogeny than by reproductive phenotype (Hypothesis 1), we performed a Principal Component Analysis (PCA) on the species-aware Variance-Stabilized Transformed (VST—see Methods) raw RNAseq counts for the 3,718 nearly single-copy orthogroups identified across all six species. We identified 81 principal components, and the first five principal components explained 96.88% of the variance in gene expression.

**Fig. 2.**
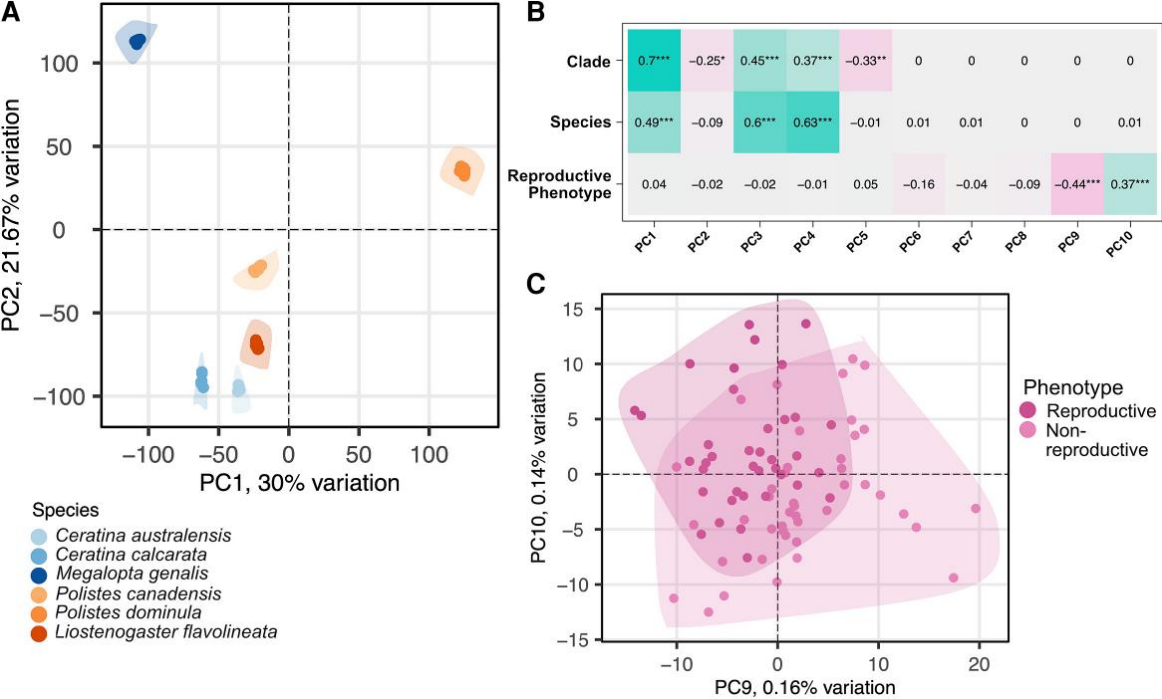
PCA shows that gene expression clusters by phylogeny rather than by reproductive phenotype.

We tested whether these PCs were significantly associated with phylogenetic clade or with reproductive phenotype. We found that across the first five principal components, phylogeny at the clade- and species-levels was significantly correlated with gene expression, accounting for 96.9% and 61.2% of the total variance in gene expression, respectively (fig. 2*A* and 2*B*). We also found PC9 and PC10 to be significantly correlated with reproductive phenotype (fig. 2*B* and 2*C*). PC9 was negatively correlated with the reproductive phenotypes (*r* = −0.44, *P* < 0.001), whereas PC10 was positively correlated with the reproductive phenotypes (*r* = 0.37, *P* < 0.001). However, these principal components only explained 0.30% the total variance. This suggests that while there may be a detectable reproductive phenotype-biased pattern of gene expression common to these species, the pattern is less robust than the effect of phylogenetic clade and may pertain to only a small fraction of genes in the transcriptome. When the first two principal components for the transformed expression values for each species were plotted, we found some species have more distinct expression patterns (e.g., *Ce. australensis*) than other species (e.g., *Polistes canadensis*) (supplementary fig. S1, Supplementary Material online). We find that the percent of variation in gene expression is explained predominantly by phylogeny, supporting Hypothesis 2. Given the limitations of PCA, namely the variables supported by the first PCs not being related to the phenotypes, we next took a three-pronged analytical approach to independently assess the strength of shared patterns of gene expression by clade and by reproductive phenotype, described below. This approach included gene differential expression (DE) analyses, a Support Vector Machine (SVM) machine learning method, and a weighted gene co-expression analysis (WGCNA).

All three panels use the 3,718 nearly single-copy orthologs across the six species. Species-aware VST RNAseq counts (which serve to normalize the data across species making them comparable) were used to find the principal components. (A) The first (PC1) and second (PC2) principal components, which cumulatively explain 51.67% of variance in gene expression. Gene expression clusters tightly by species but not necessarily by clade (bee vs. wasp). Polygons have been drawn around the two phenotypes in each species to show the lack of overlap. (B) The eigenvalue correlations of the principal components with respect to clade, species, and reproductive phenotypes were calculated using a Pearson correlation coefficient. Shown are the correlations for the top ten PCs. Color shading indicates the strength of the correlation, and asterisks indicate level of significance after Benjamini–Hochberg (Benjamini and Hochberg 1995) correction with *P < 0.05, **P < 0.01, and ***P < 0.001. (C) PC9 and P10 were significantly correlated with reproductive phenotype. The points represent the individual RNAseq samples of the six species (*n* = 81 total) separated by phenotype. PC9 was negatively correlated with reproductive phenotype, whereas PC10 was positively correlated. Note that the clustering of the genes in those principal components overlap. Polygons are drawn around the clouds of points to show the degree of overlap.

### Genes with Large Fold-change Expression Differences do not Identify Social Phenotype-biased Patterns

Previous analyses of each species’ dataset had revealed expression differences between reproductives and non-reproductives (Patalano et al. 2015; Jones et al. 2017; Rehan et al. 2018; Shell and Rehan 2019; Taylor et al. 2021). We took a two-pronged approach where we first looked for common differentially expressed (DE) genes between reproductive and non-reproductive phenotypes across the six species of bees and wasps, restricting our analysis to the 3,718 nearly single-copy orthologs. We found 197 orthologs that overlapped among two or more of the six species after controlling for species-specific expression variance (supplementary Table S19, Supplementary Material online). Notably, there were no DE orthologs found common to all six species (maximum four species). Our second approach intended to assess whether there were any functional annotations common across the six species, thereby not restricting our analysis to orthologs. We found no overlap in gene function across all six species (supplementary Table S19, Supplementary Material online). Given that DE gene analyses does not provide higher levels of gene associations, such as regulatory network and co-expressed gene networks, we then tested our hypotheses using analyses measuring subtle changes in gene expression (SVM) and gene network analyses (WGCNA).

### SVM Identifies Correlated Sets of Genes that Predict Social Phenotype Across all Species

**Fig. 3.**
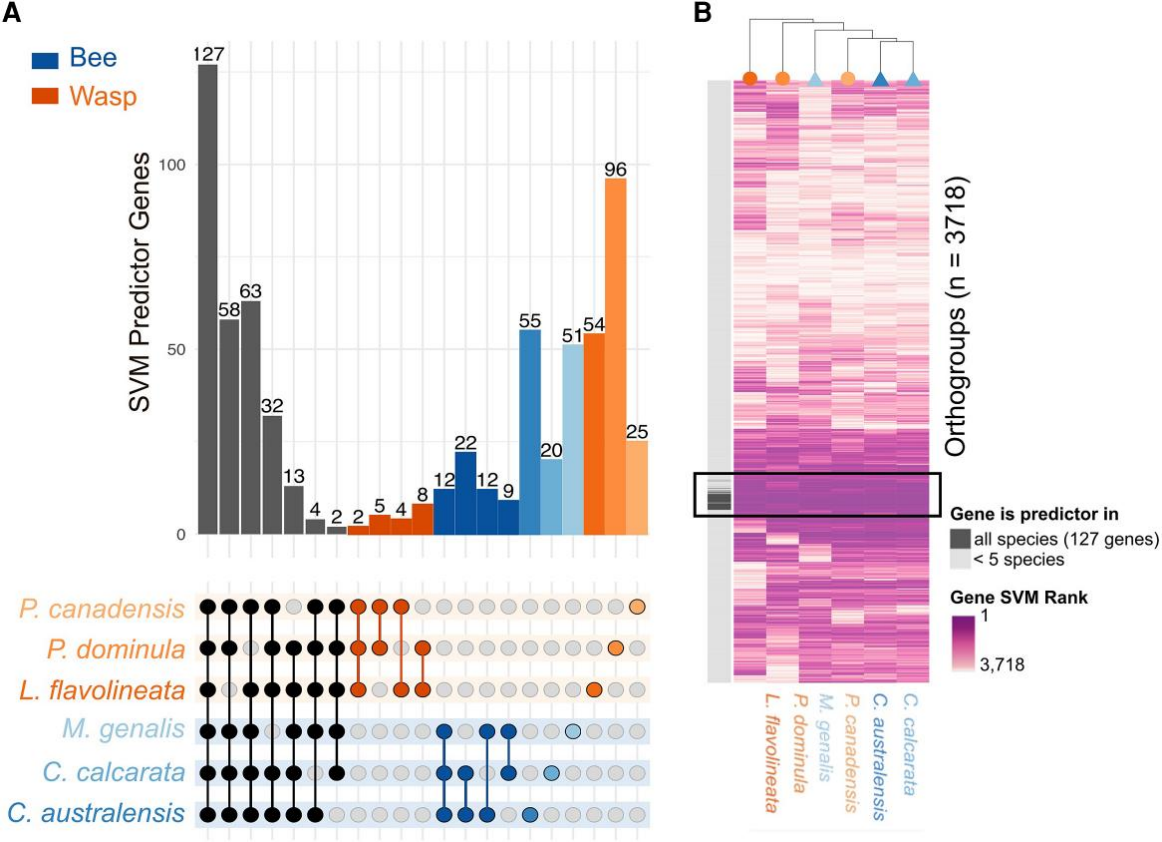
Reproductive phenotype is predicted by species-specific orthologous gene sets, including 127 genes that comprise a toolkit for rudimentary societies across all six species A) Overlapping analysis of leave-one-out SVM results, after testing one species on a training set made of the five other species. In the upper part, the bar plot shows the frequency of orthogroups (*n* = 3,718) that are significantly predicting phenotypes (reproductives vs. non-reproductives) in selected overlapping sets of leave-one-out SVM results. In the lower part, the relationship matrix shows filled circles for the species (top rows: wasps; bottom rows: bees) sharing predictor genes sets (columns). For instance, 127 orthogroups are common predictor genes to all six species (left bar), and a total of 299 are common predictor genes to at least five species (total of the first seven bars on the left). B) Orthogroup ranking in each SVM analysis clustered by Euclidean distances of 3,718 orthogroups (in rows) and six species (in columns). The darker the shade, the more important this orthogroup is in predicting phenotype (i.e., higher rank). The darker section in the vertical column on the left indicates the 127 orthogroups that are significantly predictor of phenotypes and common to all species.

Using a leave-one-species-out SVM Learning approach, we identified a different set of 127 genes with consistent shared patterns of DE among the social phenotypes of all six species of bees and wasps (fig. 3*A*, supplementary Tables S7–S9, Supplementary Material online). SVM rank of each gene (i.e., how strongly an orthogroup predicts reproductive phenotype) did not cluster species according to lineage (fig. 3*B*).

Instead, each species is unique in its prediction strength of gene expression, and branches off on its own in the hierarchical clustering, except for the two *Ceratina* bee species (sister taxa in fig. 3*B*). This could reflect the noise from all 3,718 genes, thus we focused next only on the predictor orthogroups.

Gene ontology (GO) terms associated with these 127 orthogroups included histone H3-K27 methylation (GO:0070734), sensory organ precursor cell fate determination (GO:0016360) and hormone biosynthetic process (GO:0042446) (supplementary Table S8, Supplementary Material online), although none were significantly enriched (Benjamini and Hochberg 1995). BLAST comparisons of predictor genes against protein sequences from honeybee, *A. mellifera,* revealed high similarity scores to the histone demethylase *UTY*, the *Vitellogenin precursor* gene, and a zinc finger transcription factor (supplementary Table S9*A*, Supplementary Material online). We found no significant over-representation of transcription factors among the predictor genes. Overall, the SVM results suggest the presence of a shared genetic toolkit, albeit consisting of a small (3%) number of genes (Hypothesis 1: reproductive phenotype).

We additionally explored lineage-specific SVMs to test if closely related species presented similar patterns (Hypothesis 2a: phylogenetic clade; i.e., among bees-only and among wasps-only data, supplementary Table S7, Supplementary Material online). Given the structural smaller training datasets due to the inherent number of samples in each lineage, we expect to find less predicting genes than in our whole analysis. We found 56 predictor genes common to all three species of bees (18% overlap with 127 SVM predictor genes), including Krueppel-like protein, and 148 predictor genes common to all three species of wasps (52% overlap with 127 SVM predictor genes), including zinc finger family genes (supplementary Tables S10 and S11, Supplementary Material online, respectively). Enriched GO terms included regulation of cell fate specification (GO:0042659), sex differentiation (GO:0007548) for bees; and chromatin organization involved in regulation of transcription (GO:0034401), regulation of cell fate specification (GO:0042659) for wasps (supplementary Tables S10 and S11, Supplementary Material online, respectively), although none were significantly enriched based on 5% False Discovery Rate.

### Gene Network Analyses Identify Common Modules of Genes that are Associated With Social Phenotype

To test whether patterns of co-expressed genes are conserved across our six species of bees and wasps (Hypothesis 1) or within each clade (Hypothesis 2), we constructed a multispecies co-expression network combining the data from all species and social phenotypes. We looked for the presence of a conserved network and determined whether modules within this bee + wasp gene network are significantly associated with social phenotype.

**Fig. 4.**
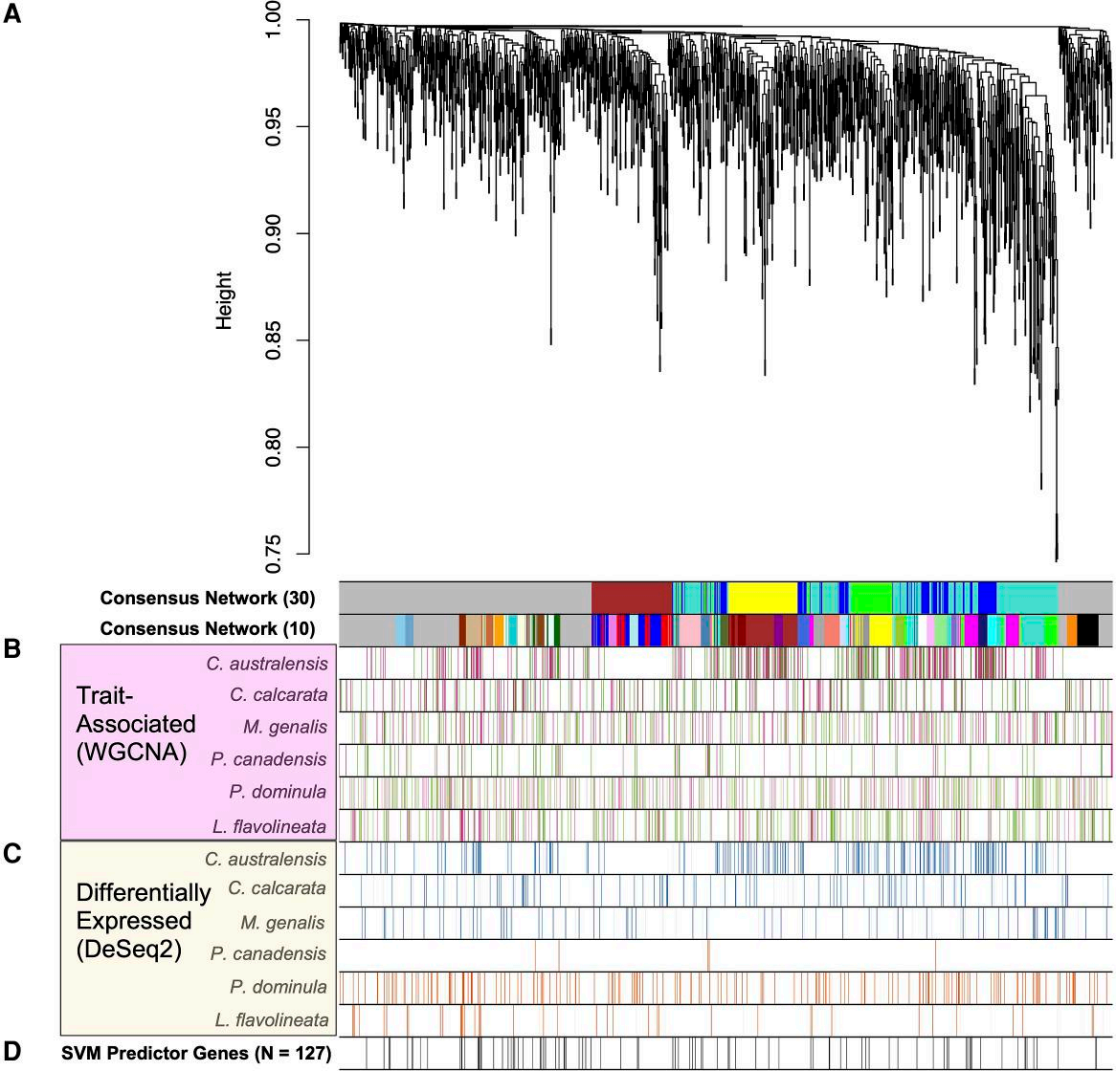
Comparing the datasets: consensus network of gene expression from reproductive and non-reproductive females’ brains in six species of bees + wasps, using nearly single-copy orthogroups. Top Panel *A*: Modules with a minimum module size of 30 genes and after relaxing to a minimum module size of 10 are shown relative to the dendrogram of 1,507 orthogroups across bees + wasps with sufficient expression for analysis. Trait-Associated (WGCNA) Panel *B*: Heatmap showing genes that are significantly phenotype-associated with reproductives (pink) and non-reproductives (green) for each species. Color strength is determined by correlation coefficient. Differentially expressed (DeSeq2) Panel *C*: Heatmap showing genes with DE in reproductives or non-reproductives for each bee (blue) and each wasp (orange) species. SVM Predictor Genes Panel *D*: The presence of the 127 genes identified by SVM as predictors of social phenotype is shown as black bars.

We found a conserved network across all six species and within clade (fig. 4*A*, supplementary Table S12*A*, Supplementary Material online). Using a minimum module size of 30 for bees + wasps combined, we found five consensus modules across the nearly single-copy orthogroups that contain a mean of 182 (ranging from 105 to 342) genes per module and mean connectivity (kME) of 0.0044 (±0.0747 SD). Of the five consensus modules identified for bees + wasps, one module (blue) was significantly associated with reproductive phenotype (supplementary Table S13, Supplementary Material online). Of the 568 genes in blue module, 137 were independently significantly associated with reproductive and non-reproductive phenotype (meta-analysis of trait association, (Langfelder and Horvath 2008) supplementary Table S13, Supplementary Material online). We further ascertained that these 137 genes significantly associated with the consensus modules were not a result of random chance by employing a resampling approach (*P* = 0.001, supplementary Table S13, Supplementary Material online, see also Supplemental Methods & Results). This suggests that the significantly trait-associated orthologs identified in consensus modules are robust, as they did not meet null expectations (supplementary Table S13*A*-*C*, Supplementary Material online).

We additionally conducted a deeper WGCNA analysis by lowering the module size (i.e., the threshold number of genes included in a given module), effectively focusing on smaller co-expression signals which are more likely to describe the evolutionary mechanisms across lineages and origins of sociality (Warner et al. 2019). We employed a novel iterative relaxation by steps of module size = 10 to allow for smaller modules of co-expressed genes containing overall more genes (Supplemental Methods, Supplementary Material online). At the smallest module size of 10, we found 39 modules with a mean size of *N* = 31 genes (ranging from 12 to 104 genes) and a mean connectivity of 0.0061 (±0.08 SD, supplementary Table S13*A*, Supplementary Material online), including 12 modules significantly associated with reproductive phenotype for a total of 1,468 genes. These phenotype-associated genes were significantly enriched for GO terms associated with chromatin such as regulation of chromatin assembly or disassembly (GO:0001672), RSC-type complex (GO:0016586), brahma complex (GO:0035060) (supplementary Table S14, Supplementary Material online). Interestingly, one of the orthogroups in the significant modules contributes to the top 10% loadings of PC9 and PC10 (fig. 2*C*), with a sequence similar to *Drosophila melanogaster*'s *small bristles* gene associated with binding activity. In summary, we found multiple genes—notably chromatin regulation—under a common co-expression pattern associated with social phenotypes across bee and wasp lineages.

### Comparing Methods Provides Robust Evidence of Shared Genetic Toolkit at the Functional Level

We compared the sets of phenotype-informative genes identified across the three methods of analyses (fig. 4: WGCNA (panel A & B); DESeq (panel C) and SVM (Panel D) to assess level of overlap and assemble a robust list of putative toolkit genes for sociality. Of these, 17 (13.4%) DEGs overlapped with the significant phenotype-associated genes from consensus WGCNA and only three (2.4%) overlapped with the 127 SVM predictor genes (supplementary Table S17, Supplementary Material online). Only two genes overlapped among all three methods: these were an orthogroup nearly matching a chitin deacetylase isoform in *Apis laboriosa* (OG0000706, BLAST nr, 99.4% identity) involved in molting and pupation in insects (Li et al. 2021), and an uncharacterized protein in honeybee (OG0001935, supplementary Table S9, Supplementary Material online). Given the small gene overlap across all three methods, we narrowed our gene set to the overall between SVM and WGCNA results.

There are 71 orthologous genes identified as significantly associated with reproductive status across all six species (fig. 4, supplementary Table S13, Supplementary Material online), present in both the SVM predictor gene set (out of 127 genes) and the consensus WGCNA (out of 1,468 genes in size-10 modules). We propose this shared gene set as a putative genetic “toolkit” for rudimentary sociality in bees and wasps. GO terms in the genetic toolkit include imaginal disc-derived wing margin morphogenesis (GO:0008587), sensory organ boundary specification (GO:0008052), neurogenesis (GO:0008052). The sequences in the genetic toolkit match honey bee annotated proteins: zinc finger protein ubi-d4 A isoform X1 (XP_395098.4), early growth response protein 1 (XP_006560759.1, also upregulated in bee foraging (Singh et al. 2018)), and an isoform of tankyrase (XP_026301139.1) involved in telomere length regulation (Bonasio et al. 2012) (supplementary Table S21, Supplementary Material online). We also looked at the UniProt functions of those 71 genes; notably four categories that have been previously associated with caste differentiation in social insects: chromatin binding (de Araujo and Arias 2021), DNA binding (Singh et al. 2018), telomere length regulation (Bonasio et al. 2012), and signal-based protein delivery (Wang et al. 2020) (Table 1 and supplementary Table S21, Supplementary Material online).

**Table 1:** Functions of the Core Genetic Toolkit Genes as Identified by GO Analyses

Number of Toolkit Genes	Summary of Functions	Notable examples and overlap with prior studies
7	Chromatin binding	AT-rich interactive domain-containing protein 2 isoform: Differentially expressed between nurses and foragers in *B. terrestris* and stingless *T. angustula* (Araujo and Arias 2021)
9	DNA binding	early growth response protein 1: Upregulated in honeybee foraging (Singh et al. 2018)
1	Regulation of telomere length	tankyrase isoform: Conserved differentially methylated gene between queens and workers in the ants *Ca. floridanus* and *H. saltator* (Bonasio et al. 2012)
1	Delivery of cellular proteins responding to a signal	thyroid receptor-interacting protein 11 isoform: Differentially methylated gene between honeybee castes (Wang et al. 2020)
12	Cellular functions	Examples: transport of ion (sodium channel protein 60E isoform X1), lipid (nose resistant to fluoxetine protein 6 isoform X2), protein (exportin-7 isoform); carbohydrate and glucose metabolic processes (beta-galactosidase)
3	Developmental processes	Examples: anatomical structure morphogenesis (carboxypeptidase M isoform), regulation of developmental processes (ubiquitin carboxyl-terminal hydrolase 34)
19	Basic processes	Non-exhaustive list: Protein modification (kelch-like protein diablo); RNA binding (poly(A) RNA polymerase gld-2 homolog A isoform); Cell cycle (tetratricopeptide repeat protein 28 isoform); Uncharacterized proteins (*n* = 12)

**Fig. 5.**
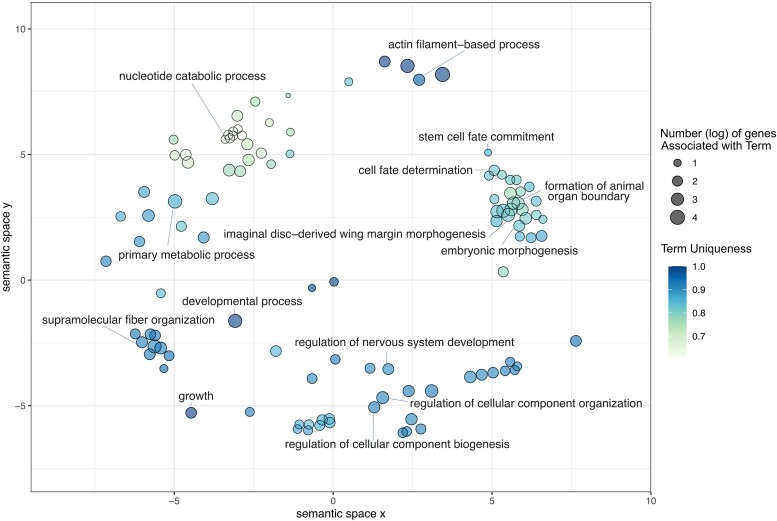
Semantic similarity of GO Terms from genes in the shared genetic toolkit.

We further conducted a semantic similarity search of the enriched GO Terms that were overlapping between the genes identified using WGCNA and SVM, using Revigo's semantic similarity index (Supek et al. 2011). Shared GO terms were related to chromatin, such as supramolecular fiber organization; telomeres, such as nucleotide catabolic process; and caste differentiation, such as cell fate determination (fig. 5).

Revigo Semantic-similarity-based multidimensional scaling of Biological Processes enriched GO Terms common to consensus WGCNA and species-normalized SVM. Each point represents a GO Term with a dispensability score below 0.15. The darker the point is, the more semantically unique the GO term is. A larger point represents a GO Term common to a large number of genes in the shared genetic toolkit.

Summary description of the genes that are common to the SVM predictor list and to the WGCNA modules associated with reproductive status (52 are characterized, 19 are uncharacterized; see full report in supplementary Table S21, Supplementary Material online).

## Discussion

Deciphering the building blocks of social behaviors in the rudimentary societies offers novel insights into the proximate and ultimate processes of social evolution (Berens et al. 2015; West et al. 2015). Despite enormous interest in the behavior, ecology and evolution of bees and wasps that exhibit such rudimentary styles of sociality (Patalano et al. 2015; Shell et al. 2021), data on their lives in molecular terms have been sorely lacking. The last few years have seen some redress to this, but comparisons across datasets have proved challenging and limited largely to comparisons of gene lists (Kapheim et al. 2020; de Araujo and Arias 2021; Korb et al. 2021). The current study sought to move beyond this, to conduct a controlled, comparative meta-analysis of six species with rudimentary sociality, spanning multiple taxonomic lineages (bees and wasps) and origins of sociality. Using a combination of several analytical approaches, this study presents the most comprehensive and robust test to date of the contributions of shared molecular mechanisms to shaping social phenotypes in the early stages of social evolution.

Previous analyses were limited to post-hoc comparisons of gene lists derived from datasets that have been analysed using different pipelines and methods. However, such comparative studies also present several challenges. Comparisons between datasets are rarely quantitative meta-analyses where collated data from different studies are reprocessed and reanalyzed in a standardized manner. This is especially important for transcriptomic datasets as sequencing methodologies have changed substantially over the last few years (Todd et al. 2016); the quality (e.g., coverage), type of data (e.g., length of reads) and tissue type (e.g., brain vs. whole head or body) may influence the robustness of the comparisons being made. Even more challenging is that the integrity of bioinformatic methods has changed greatly over time, from upstream pipelines that deal with the raw sequencing data, assembly programs and annotation databases, to down-stream computational methods that compare levels of gene expression, functional enrichment and how genes relate in networks.

Our analysis utilized transcriptomic datasets of three wasp and three bee species, displaying a variety of rudimentary forms of social organization, but all characterized by being comprised a small number of non-reproductive adults nesting with one reproductive. In addition to traditional analyses (differentially expression), we employed co-expressed gene network and unsupervised statistical machine learning methods (SVM), and successfully unveil a common set of genes and molecular functions for this cluster of species at the emergence of social group living. We found that traditional methods (using statistical cut-offs of significance based on DE) failed to identify cross-species similarities; this suggests a more nuanced and sensitive approach is needed to identify gene expression similarities across such a wide evolutionary distance. The application of machine learning methods is relatively new to analyses of non-medical genomic data (Wyatt et al 2020; Taylor et al 2021); using SVMs we were able to identify a set of 127 genes that consistently performed well in the classification of social phenotypes from their gene expression across all six species. A comparison of this trio of analytical methods allowed us to identify a conserved set of genes related to reproductive and non-reproductive social phenotypes in rudimentary societies across lineages and life histories. This represents a putative shared genetic toolkit for the early stages in the evolution of a reproductive division of labor—a hallmark of eusociality.

Social phenotypes were not the only factor explaining patterns of brain transcription: we found a strong phylogenetic signal, whereby each bee and wasp lineage showed taxon-specific patterns of brain gene expression. In fact, the primary factor clustering differentially expressed genes among our samples was taxonomic group (fig. 2). This is not surprising given that these species represent 200 Myr of divergence, and various degrees and forms of social organization across species (fig. 1); although, some of these patterns could be due to differences in diet as bees are pollen collectors whilst wasps are predators. The impact of lineage-specific selection pressures, associated with both independent evolution of social organization and emergence of novel genes, has previously been highlighted in comparative transcriptomics analyses between Hymenoptera lineages (Berens et al. 2015; Warner et al. 2019; Kapheim et al. 2020).

The genetic toolkit empirical hypothesis for the evolution of sociality posits there is an evolutionarily conserved mechanistic trajectory to social organization across taxa and social origins. It predicts deeply conserved common individual genes, or shared co-expressed gene network modules to be found as differentially expressed in convergent social forms across taxa. The null hypothesis proposes no detectable similarity in the expression of genes or modules related to alternative social phenotypes shared across rudimentary social species. The core set of genes differentially expressed between alternative social phenotypes we identified here through multiple methods suggest that there may be similar molecular functional changes across rudimentary social insect societies. We identified conserved functions in chromatin binding, which has been observed differentially expressed between nurses and foragers in the eusocial bees *Bombus terrestris* and stingless *Tetragonisca angustula* (Araujo and Arias 2021) (Table 1). We also identified conserved functions in DNA binding, which is upregulated in honeybee foragers (Singh et al. 2018), and regulation of telomere length, which has been shown to be differentially methylated between queens and workers in ants *Camponotus floridanus* and *Harpegnathos saltator* (Bonasio et al. 2012). Lastly, we see genes related to reproduction (e.g., yolk protein vitellogenin) and gene functions related to metabolism (e.g., carbohydrate and lipid metabolism), which have been consistently implicated as key players in a conserved genetic toolkit for sociality (Toth and Robinson 2007; Amsalem et al. 2014; Berens et al. 2015; Morandin et al. 2019). These results—71 core genes—are within the same range as other studies on conserved expressed genes between: ant slave-making species and host species (*n* = 62 genes shared across 162 Ma of divergence [Feldmeyer et al. 2022]); animal behavior after social challenge (*n* = 6 genes shared across 680 Ma of divergence [Saul et al. 2019]). Overall, the emerging picture from our and these studies is that there are conserved gene modules recruited during eusocial evolution for the regulation of convergent social traits (e.g., castes), but few genes are individually predictive of a given trait. The involvement of suites of interacting gene modules is not unexpected, given the complexity of social traits (i.e., castes involve coordinated suites of behavioral, physiological, and developmental differences). Thus, unlike the classic evo-devo toolkit, the molecular toolkit for insect sociality may be more “loosely” structured around gene modules and functions, and thus detectable with more nuanced approaches such as the machine learning approach used here.

To conclude, this study used a robust multi-pronged meta-analysis, to identify a core set of genes that are consistently associated with the social phenotypes typical of rudimentary group living in the Hymenoptera. These are a putative genetic toolkit for the early stages of social evolution, laying the foundations for the emergence of a key hallmark of eusociality—a reproductive division of labor. This toolkit has been hypothesized to form the mechanistic basis upon which more derived forms of eusociality may have arisen through evolution (Toth and Rehan 2017; Wyatt et al. 2020). We encourage adoption of such multi-pronged analytical approaches in future studies that include more origins of sociality, a wider range of forms of social living, and across larger genomic datasets. Such studies will allow us to understand whether the genetic toolkit uncovered here is also related to the elaboration of sociality and the development of superorganismality.

## Materials and Methods

### Datasets

Datasets and phenotypic comparisons for the three bee species (*Ce. australensis, Ce. calcarata, M. genalis*) and three wasp species (*Polistes canadensis, P. dominula, L. flavolineata*) are given in supplementary Table S2, Supplementary Material online. RNAseq raw reads were downloaded from NCBI (supplementary Table S3, Supplementary Material online). For the reproductive and non-reproductive phenotypes, sample sizes ranged from three to 12 individual whole-brains (except for *Ce. calcarata*, which were individual whole heads). Raw read depth per sample ranged from 17,453,576 to 96,115,726 (mean 32,514,855.6 ± 13,571,313.98 SD) (supplementary Table S2, Supplementary Material online). All scripts are available on GitHub: https://github.com/EmelineFavreau/MajorTransitionScripts/tree/master/comparative-transcriptomics.

### Orthogroup Identification

Prior to orthogroup identification, we assessed completeness of the predicted genes and longest-isoform protein sequences for each of the six species with BUSCO using the Arthropoda and Hymenoptera lineage reference datasets, respectively (supplementary Tables S2 and 3, Supplementary Material online) (Simão et al. 2015). We then obtained orthologous gene sets using OrthoFinder v. 2.4.0 (Emms and Kelly 2019) using *A. mellifera* (honeybee) as an outgroup. Because bees and wasps diverged nearly 200 Ma (Peters et al. 2017), and divergence times within the bees and wasps are 90–100 Ma and 145–167 Ma (Branstetter et al. 2017; Peters et al. 2017), respectively, we used a relaxed filtering approach for orthogroup identification. We allowed between one and three gene copies per species and for an orthogroup to be absent in up to one species (supplementary Table S6, Supplementary Material online).

### Brain Transcriptome Read Mapping

We reprocessed all RNAseq raw reads in a standardized way using the publicly available Nextflow wrapper nf-core/rnaseq v.1.4.2 (di Tommaso et al. 2017). In short, for each dataset, we trimmed raw RNAseq reads with TrimGalore! (Krueger et al. 2021), mapped the reads to their respective genome with STAR (Dobin et al. 2013), and obtained GFF (gene_id) feature read counts with FeatureCounts (Liao et al. 2014) (supplementary Table S5, Supplementary Material online). Read directions were adjusted as needed per experiment. We assessed mapping quality to ensure similarity across the six species’ datasets (Supplemental Methods and Results). Throughout, we refer to annotated features from the GFFs as “genes’.

### Pre-processing Transformation of Data

Fair comparison of features between species, such as DEGs and SVM, requires degrees of pre-processing transformations. We employed three methods: variance stabilization, species normalization (‘species awareness’), and data scaling, which we describe here. Prior to all analyses, combined raw read counts of all species and samples were transformed by variance stabilization using the *VST* function in DESeq2 (Love et al. 2014). This generated constant variances within the matrix of all read counts (i.e., a homoscedastic dataset). Where the experimental design called for “species awareness’, that is controlling for the effect of species on gene expression, we included species as an explanatory variable in the model when constructing the matrix of VST read counts. This generated species-aware data. Finally, SVM analyses require center-scaled data to make each sample comparable to another. This was accomplished by setting the mean of the species-aware VST counts to zero.

### Principal Component Analysis

We conducted a PCA to examine whether overall brain gene expression clusters more by phylogeny than by reproductive division of labor as hypothesized. We performed the PCA using the PCAtools package in R (Blighe and Lun 2021) on the VST (Love et al. 2014) raw RNAseq counts for 3,718 nearly single-copy orthogroups identified across the six species. We favored PCA over clustering methods such as cancer single-cell RNAseq autoencoders (Eraslan et al. 2019), because our sample size is smaller than typical cancer single-cell RNAseq. A PCA is an unsupervised method used to reduce the dimensionality in large datasets by taking linear combinations of data—here gene expression—to define a new set of uncorrelated variables, called principal components. PCs are ordered to capture the maximum variance explained, and thereby can be used to automatically define PCs that explain the most variation possible in gene expression. We took the top five PCs and tested individual eigenvalues for correlations with our traits of interest: clade, species, and reproductive phenotype. We then used PC biplots to infer the distances between samples based on their gene expression.

We also repeated each PCA on each species for all genes with an RNAseq transcript > 1, separating samples by whether they come from reproductive versus non-reproductive samples (supplementary fig. S1, Supplementary Material online) and found that gene expression patterns are, in some species more than in others, distinct between alternative social phenotypes. Finally, we ran randomized PCA by shuffling the attributed phenotypes and confirmed that the subtle differences between gene expression cannot be solely measured by PCA (supplementary fig. S23, Supplementary Material online).

### Machine Learning Analyses with SVM

SVM (Cortes and Vapnik 1995) is a supervised classification algorithm that can be used to predict phenotypes on the basis of data classification such as morphological measurements or expression patterns. It has been used to distinguish subtle differences in human cancer subtypes (Yuan et al. 2020), to find non-expressed yet cancer-associated genes (Ghanat Bari et al. 2017) and to explore microbiome (Dhungel et al. 2021); as well as to contrast behavioral phenotypes in social Damaraland mole-rats (Johnston et al. 2021), in honeybees (Liang et al. 2014), and paper wasp *P. dominula* (Taylor et al. 2021). SVM is a complementary approach to conventional differential gene expression analysis such as DESeq2. It has proven successful classification accuracy in benchmarks study against other methods such Random Forest (Zararsz et al. 2017) and Naïve Bayes Classifier (Ramdaniah et al. 2019) and has proven equally successful as Random Forest in predicting waggle dance genes (Veiner et al. 2022).

SVMs identify the hyperplane between classes such that the distance between the hyperplane and the nearest point of the classes has been maximized. Even when there is no separating hyperplane, SVM produces classifiers by allowing some classification error up to a constant and maximizing the margin between the hyperplane and the nearest point. This may further make it a suitable choice for distinguishing subtle differences in gene expression that may be less likely to be detected by conventional DE methods due to either low sample sizes or noisier expression patterns, as seen in plastic phenotypes (Taylor et al. 2021) or *in-silico* pooled data containing both septic and non-septic patient samples (Schaack et al. 2021).

We use a Train/Test Split approach, in which we test the data of each species against a model that has been trained on the other five species’ datasets (see schematic in supplementary fig. S21, Supplementary Material online). The result is a list of the 3,718 nearly single-copy orthologous genes predicted by the SVM as differentially expressed between reproductive (coded as *1*) and non-reproductive (coded as *0*) phenotypes.

In short, the raw read counts of the 3,718 orthologous genes in reproductive and non-reproductive samples from the six species (82 samples in total) were first transformed by variance stabilization with use of the full experimental design (i.e., within species normalization) in the DESeq2 R package (Love et al. 2014; R Core Team 2014). The data matrix was then center-scaled. To identify the appropriate kernel function, we calculated accuracy rates from SVM models run on each dataset using the e1071 R package (Meyer et al. 2015) with linear and radial kernels and the following parameters: formula = phenotype ∼ read counts, type = C-classification. Radial kernel consistently led to better prediction accuracy (i.e., higher accuracy rate for radial kernel than linear kernel, see confusion matrices in supplementary Table S7, Supplementary Material online and Receiver Operating Characteristics curves in supplementary fig. S20, Supplementary Material online). Thus, all subsequent models were fit using this kernel and a grid search of gamma between 10^−7^ and 10^−5^ and cost between 2^3^ and 2^5^. Next, for each of the six species, we constructed a full model with a *k* = 3-fold cross validation, in which a random third of the training samples is tested against the remaining training samples. Next, for each of the six species, we constructed a full model with a *k* = 3-fold cross validation, in which a random third of the samples for that species is tested against the remaining all species’ samples as training data. K-fold Cross-Validation technique was recently benchmarked the best for sample size range of 20–100 (Vabalas et al. 2019). We thus obtained a full-model prediction error rate for each species based on the predicted phenotype versus the actual phenotype. Error rate is the performance measure of the prediction, specifically the mean squared error rate for regression (from the e1071 R package tune function): the smaller the error rate is, the better the SVM predicts the phenotype based on the read counts.

We then opted for feature selection (i.e., filtering for the genes that best predict the phenotype) as an embedded method in the SVM iterations, because it is fast, better performing than univariate filter techniques (Saeys et al. 2007), and widely used in detection of loci associated with cancer (Abeel et al. 2010), plant drought-resistance (Liang et al. 2011), honey bee waggle dance (Veiner et al. 2022). We performed a leave-one-species-out iterative process, in which one species was chosen as the test dataset and the remaining five were used as the training set. Over 20 iterations, SVM models were run while fine-tuning the parameters of gamma and cost. The resulting model with the lowest error rate was used to assign weights to each of the 3,718 genes, where a higher weight meant that the gene was better at predicting the reproductive phenotype (coded as *1*). Feature weights were calculated by taking the matrix product of the coefficients for the model with its support vectors. We then performed recursive feature elimination, where at each step the gene with the lowest weight was removed, and the resulting model was again tuned using the remaining genes. This remove-one-gene cycle was iterated until the input dataset contained 100 genes only, when the error rate trend increases as seen in a previous study (Taylor et al. 2021). We selected the optimized model with the lowest error rate amongst these 3,618 best-performing models. To validate the models, we ran additional randomization tests in which focal species’ phenotypes were shuffled prior to be tested by the trained SVM model. We ran this randomization 100 times for each focal species, and assessed the true error rate against the distribution of error rates from randomized tests (supplementary fig. S22, Supplementary Material online). Lastly, a final SVM model was run using the parameters and the gene predictors of the optimized model. This resulted in a set of gene predictors that represent DE genes between the reproductive and non-reproductive phenotypes for each of the six species. We then further filtered these predictor genes for those that overlap (1) across all six species and (2) overlap within just the bees or just the wasps. We also ran SVMs within a given lineage: testing a bee against the two other bees, and a wasp against the two other wasps.

### Weighted Gene Co-expression Network Analysis

We used two conceptual frameworks to compare gene co-expression network conservation across all six species of bees and wasps, as well as within each clade. First, a multispecies co-expression network was constructed using nearly single-copy orthogroups to provide an overarching view of network structure for the species compared (i.e., orthology-dependent). We used this to ask whether a conserved network of gene expression exists, and whether any modules within that network were significantly associated with social phenotype across all species compared. Second, an individual species network was constructed from all genes with sufficient expression for that species, regardless of orthology (i.e., orthology-independent). All analyses were performed using the “wgcna” package (Langfelder and Horvath 2008) on variance-stabilized (Love et al. 2014) read counts after removing genes with zero expression or without variance across samples.

First, we constructed consensus co-expression networks using variance-stabilized read counts with Weighted Gene Co-expression Network Analysis (WGCNA). We manually constructed consensus networks using the nearly single-copy orthogroups shared across the species in the comparison. The resulting consensus network modules are based on the co-expression distance after hierarchical clustering. We then performed a meta-analysis testing for significance of network modules with reproductive phenotype and report those genes with significant membership in the phenotype-correlated modules and which show significant correlation with the phenotype themselves. We calculate a Z-score to test the module preservation relative to our phenotypes of interest, as well as to test whether specific genes are significantly correlated across all species with (a) phenotype-associated modules and (b) reproductive status regardless of module status (Langfelder et al. 2013). We further tested whether significantly trait-associated orthologs were a result of random chance and small sample sizes by testing whether they deviate from a null expectation. To do this, we shuffled the reproductive status labels before constructing the network, and resampled the network *k* = 1000 times. We calculated the proportion of point estimated genes found in a given resampling event, and derived a two-tailed *P*-value as the number of times this proportion was more extreme than 50%, the null expectation, divided by *k* resampling events.

Note that the topology of the network is highly dependent on the minimum module size set in WGCNA; therefore, we also took an iterative approach to the minimum module size parameter. We report results for a minimum module size of 30 and 10, as 10 was found to be robust for module preservation in a benchmarking study (Li et al. 2015).

Second, we constructed individual co-expression networks from variance-stabilized read counts of all genes with minimal expression regardless of homology. The genes that were significantly correlated with reproductive and non-reproductive phenotypes were then used to test for common functional enrichment among the species.

### Differential Gene Expression Analysis

To compare with the results of the SVM, differentially expressed genes were called between reproductive and non-reproductive phenotypes in two ways: orthology-dependent and orthology-independent. The orthology-dependent analyses were intended to compare the results of DE gene calling to those of the SVM. We also opted for an orthology-independent analysis to identify possible non-orthologous differentially expressed (DE) genes. For example, a common toolkit across these six species might not be conserved genes but instead conserved functions.

For the orthology-dependent analysis, for each species we used DESeq2 (Love et al. 2014) to call DE genes between reproductive and non-reproductive phenotypes. In this method, we applied the DESeq function to the raw read counts for the nearly single-copy orthogroups for the species comparisons being made (all six species, bees only, wasps only). Significant differential gene expression was determined using an FDR-adjusted *P*-value of 0.05 as the threshold. For the orthology-independent analysis, the raw read counts for all genes for each species in the comparison were used. We also employed a permutation-testing approach to ensure that there was no difference in the numbers of DE genes obtained from DESeq2 due to uneven sample sizes among our species (supplementary Table S6, Supplementary Material online). The DESeq function was applied only once using a model that included a term for clade in addition to phenotype, and significant DE genes were called using an FDR-adjusted *P*-value of 0.05. This was to allow us to identify any genes with a sufficiently strong signal of DE across all species in the comparison after accounting for phylogeny.

For the orthology-independent analysis, we took all raw read counts for each species without filtering for orthogroups. For each species, we identified which genes are significantly DE between the reproductive phenotypes using an FDR-adjusted *P*-value of 0.05. We once again employed a permutation approach to correct for differences between the species in the number of samples.

### Functional Interpretation of Candidate Genes

We performed GO Term enrichment analysis for genes that are significantly DE. We first obtained the best similarity hits against *D. melanogaster*'s protein set using BLASTp V.2.2.30 (Altschul et al. 1990) for each species’ protein set. We then obtained enriched GO Terms using R biomaRt v. 2.42.1 (Durinck et al. 2005) and TopGO v. 2.38.1 (Alexa and Rahnenfuhrer 2010) with the following parameters: terms with at least five annotated genes, classic algorithm, Fisher statistics. We find between 39 and 206 enriched Biological Processes GO Terms per species, between 18 and 72 enriched Molecular Function GO Terms, between 4 and 68 enriched Cellular Components GO Terms (Table ST9). Only a few GO Terms are common to several species, the largest set is 15 GO Terms common to *Ce. australensis* and *L. flavolineata*. We also performed GO Term enrichment analysis for WGCNA genes (supplementary Table S10, Supplementary Material online) and SVM predictor genes (supplementary Tables S4, S16, and S17, Supplementary Material online).

We performed a REVIGO analysis of the enriched GO terms from 127 SVM predictor genes (parameters: medium resulting list, species: Drosophila melanogaster, SimRel measure [Supek et al. 2011]), reducing the GO Term list to less redundant Terms (fig. 3).

## Supplementary Material

evac174_Supplementary_Data

## Data Availability

The data underlying this article are available in NCBI at https://www.ncbi.nlm.nih.gov/, and can be accessed with PRJNA406923 (*Ce. australensis*), PRJNA434715 (*Ce. calcarata*), PRJNA801104 (*L. flavolineata*), PRJNA331103 (*M. genalis*), PRJNA255520 (*Polistes canadensis*), and PRJNA642907 (*Polistes dominula*).
