# Supplementary material for: Co-expression Gene Networks and Machine-learning Algorithms Unveil a Core Genetic Toolkit for Reproductive Division of Labour in Rudimentary Insect Societies
Source: Genome Biol Evol. 2022 Dec 17;15(1):evac174. doi: 10.1093/gbe/evac174 (PMC9830183; doi:10.1093/gbe/evac174)
Supplement: evac174_Supplementary_Data [file evac174_supplementary_data.zip › GBE-220429-R2-Supplementary Information.docx]

*Supplementary Information for GBE submission (Research Article)*

Title: Co-expression gene networks and machine-learning algorithms unveil a core genetic toolkit for reproductive division of labour in rudimentary insect societies

Emeline Favreau*^1^, Katherine S. Geist*^2^, Seirian Sumner**^1^, Amy L. Toth**^2^, Sandra M. Rehan**^3^,

[Materials and Methods 3](#_Toc115256576)

[Genome Completeness 3](#_Toc115256577)

[Transcriptome Quality 3](#_Toc115256578)

[Differential Expression (DE) Analyses 3](#_Toc115256579)

[DESeq2: Orthology-dependent approach 4](#_Toc115256580)

[DESeq2: Orthology-independent approach 5](#_Toc115256581)

[Machine Learning Analyses with Support Vector Machine (SVM) 5](#_Toc115256582)

[Weighted Gene Co-expression Network Analysis (WGCNA) 7](#_Toc115256583)

[Multispecies Consensus WGCNA 7](#_Toc115256584)

[Orthology-Independent (Individual Species) Coexpression Networks 8](#_Toc115256585)

[Functional Interpretation of Candidate Genes 9](#_Toc115256586)

[**Results** 10](#_Toc115256587)

[Transcriptomes are of similar quality and completeness for downstream analyses. 10](#_Toc115256588)

[Minimal commonly differentially expressed (DE) genes between alternative social phenotypes using conventional analyses 10](#_Toc115256589)

[Orthology-dependent approach reveals no commonly differentially expressed genes between alternative social phenotypes either across bees and wasps or within each clade 11](#_Toc115256590)

[Orthology-independent approach reveals no shared functions of differentially expressed genes between alternative social phenotypes either across bees and wasps 12](#_Toc115256591)

[Support Vector Machine analysis finds a small number of genes with conserved caste-biased expression between bees and wasps, as well as within clade. 13](#_Toc115256592)

[Consensus WGCNA identifies a different, but overlapping, set of caste-biased genes from SVM 14](#_Toc115256593)

[Individual species’ coexpression networks reveal no shared patterns of gene functional enrichment 15](#_Toc115256594)

[Transcription factor analyses 15](#_Toc115256595)

[Semantic similarity in GO Terms enriched in all methods (SVM, DEGs, WGCNA) 16](#_Toc115256596)

[Figures 17](#_Toc115256597)

[Supplementary Figure 1. Principal Component plots for the expression data showing Reproductive (R) vs. non-reproductive (NR) phenotypes for the 6 species in this study. 17](#_Toc115256598)

[Supplementary Figure 2. No expression bias in the 3,718 near single-copy orthogroups common to the three bee and three wasp species used in this study. 18](#_Toc115256599)

[Supplementary Figure 3. Effect of restricted (N = 3) sample sizes in identification DE orthogroups in species with N > 3 RNAseq samples. 19](#_Toc115256600)

[Supplementary Figure 4. Effect of restricted (N = 3) sample sizes in identification DE genes in species with N > 3 RNAseq samples. 20](#_Toc115256601)

[Supplementary Figure 5. SVM prediction of *Ceratina australensis* phenotype using a large training set. 21](#_Toc115256602)

[Supplementary Figure 6. SVM prediction of *Ceratina calcarata* using a large training set. 22](#_Toc115256603)

[Supplementary Figure 7. SVM prediction of *Megalopta genalis* using a large training set. 23](#_Toc115256604)

[Supplementary Figure 8. SVM prediction of *Polistes canadensis* using a large training set. 24](#_Toc115256605)

[Supplementary Figure 9. SVM prediction of *Polistes dominula* using a large training set. 25](#_Toc115256606)

[Supplementary Figure 10. SVM prediction of *Liostenogaster flavolineata* using a large training set. 26](#_Toc115256607)

[Supplementary Figure 11. SVM prediction of *Ceratina australensis* using a lineage-specific training set. 27](#_Toc115256608)

[Supplementary Figure 12. SVM prediction of *Ceratina calcarata* using a lineage-specific training set. 28](#_Toc115256609)

[Supplementary Figure 13. SVM prediction of *Megalopta genalis* using a lineage-specific training set. 29](#_Toc115256610)

[Supplementary Figure 14. SVM prediction of *Polistes canadensis* using a lineage-specific training set. 30](#_Toc115256611)

[Supplementary Figure 15. SVM prediction of *Polistes dominula* using a lineage-specific training set. 31](#_Toc115256612)

[Supplementary Figure 16. SVM prediction of *Liostenogaster flavolineata* using a lineage-specific training set. 32](#_Toc115256613)

[Supplementary Figure 17: Heatmap of 3,718 Orthologous Genes Shows that Overall Topology Differs from that Obtained by SVM Ranking. 33](#_Toc115256614)

[Supplementary Figure 18. Heatmap of 3,718 Orthologous Genes Shows that Overall Topology Differs from that Obtained by SVM Ranking. 34](#_Toc115256615)

[Supplementary Figure 19. REVIGO semantic plot of GO terms common to SVM, WGCNA and DEG analyses. 35](#_Toc115256616)

[Supplementary Figure 20. Receiver Operating Characteristics curves of SVM models testing samples from one species against samples from the five other species. 36](#_Toc115256617)

[Supplementary Figure 21. Support Vector Machine and Feature Selection Model Flowchart 37](#_Toc115256618)

[Supplementary Figure 22. SVM Randomization Tests 38](#_Toc115256619)

[Supplementary Figure 23. PCA Randomization Tests 39](#_Toc115256620)

**Materials and Methods**

#### Genome Completeness

All genomes were previously published (Supplementary Table 2), but they had not been assessed for equivalent completeness. Using the predicted genes from the reference genomes, we assessed completeness for each of the six species with BUSCO using the Arthropoda lineage dataset (odb10) as reference (Supplementary Table 3, [(Simão et al. 2015)](https://paperpile.com/c/zTstmx/Puxh4). We further assessed completeness of the longest-isoform protein sequences for each of the six species with BUSCO using the Hymenoptera lineage dataset as reference (Supplementary Table 3). The motivation was to demonstrate that, despite the deep divergence times across the six species (Figure 1), genome completeness at the Arthropod and Hymenoptera lineage levels were sufficient to allow identification of orthogroups across the six species.

#### Transcriptome Quality

We assessed whether transcriptome quality was similar across the six species, given that these were drawn from different experiments, sample sizes, time points, and even sequencing platforms (Supplementary Table 4). We compared the proportion of uniquely mapped reads, total numbers of mapped features (alignments assigned to a GFF feature), total Fragments Per Kilobase of transcript per Million (FPKM) mapped reads were similar both overall and for each phenotype, reproductive (R) and non-reproductive (NR). Hereafter, we refer to the annotated features from the GFFs that are mapped to transcripts as ‘genes’.

#### Differential Expression (DE) Analyses

We took a two-pronged analytical approach where we asked whether there are common differentially-expressed (DE) genes across the six species of bees and wasps or, if due to deep evolutionary divergence, common functions of DE genes. To ask the former question, we took an orthology-dependent approach, which compared orthologous DE genes across bees + wasps, as well as bees and wasps independently. We then looked for overlapping DE genes as well as gene functions. For the latter approach, we performed species-specific DE analyses, aggregated DE genes, and then looked for common functional annotations across those DE genes pooled across the six species.

##### DESeq2: Orthology-dependent approach

We tested whether there are genes commonly differentially expressed (DE) between reproductive and non-reproductive phenotypes using the raw read counts for the 3,718 nearly single-copy orthogroups across the six species. For the bees- and wasps-only comparisons, 5,787 and 6,983 single-copy orthogroups were used, respectively. From these, we identified DE genes between reproductive and non-reproductive phenotypes with DESeq2 [(Love, Anders, and Huber 2014)](https://paperpile.com/c/zTstmx/skuKV) for each species separately. We applied the DESeq function to the raw read counts for the nearly-single copy orthogroups for the species comparisons being made (all six species, bees only, wasps only), controlling for species in the model design. In short, the DESeq function models normalized gene counts with a negative binomial generalized linear model to correct for both variation in read sequencing depth and inter-library dispersion. Significant differential gene expression was determined using an FDR-adjusted p-value of 0.05 as the threshold.

The number of samples can influence the number and identity of DE genes obtained from an experiment [(Baccarella et al. 2018)](https://paperpile.com/c/zTstmx/d4XJ), and small sample sizes can reduce the power to detect significant DE genes between samples with DESeq2. Therefore, we employed a permutation-testing approach to ensure that there was no difference in the numbers of DE genes obtained from DESeq2 due to uneven sample sizes among our six species. For *M. genalis, L. flavolineata,* and both *Polistes* spp., we resampled *N* = 3 sample replicates from each phenotype for a total of six samples. This was to mirror the limited sample sizes of three reproductive and three non-reproductive samples available for the two *Ceratina* spp. We iteratively resampled *N* = 3 reproductive and non-reproductive samples from those four species *k* times, each time re-running the DESeq function. This allowed us to calculate the probability that the proportion of DE genes obtained on the full dataset ($\hat{m}$) differs from that if we only had three samples of each phenotype. This probability estimate, or one-sided p-value, was calculated as the number of times that the proportion *m* of DE genes is more extreme than the original proportion for the full dataset, $\hat{m}$*,* divided by 1000*.*

*One-sided p-value*

$$=\frac{\sum_{i=1}^{k} m> \hat{m}}{k}$$

Where *m* is the proportion of DE genes for for the *i* ^th^ sample, $\hat{m}$ is the point estimate for the proportion of DE genes calculated on the full number of samples for a given species, and *k* is the number of iterations, here 1000. Thus, the P-value estimated here allows us to test the null hypothesis that the point estimate is no different with all samples used if only N = 3 samples are used and determine whether sample size limited or altered our ability to detect DE genes in the two *Ceratina* spp.

We repeated the analyses for bees- and wasps-only comparisons, using 5,787 and 6,983 single-copy orthogroups, respectively. We again identified DE genes between reproductive and non-reproductive phenotypes with DESeq2 [(Love, Anders, and Huber 2014)](https://paperpile.com/c/zTstmx/skuKV) for each species separately with the DESeq function. However, permutation tests were not repeated for the bees- and wasps-only comparisons.

##### DESeq2: Orthology-independent approach

Raw RNAseq read counts for each species were analyzed for differential expression (DE) between the Reproductive (R) and Non-reproductive (NR) phenotypes with DESeq2 [(Love, Anders, and Huber 2014)](https://paperpile.com/c/zTstmx/skuKV). The proportion of genes DE in each species’ transcriptome was calculated, where the 'transcriptome' encompasses all genes with > 1 RNAseq reads. The proportion of DE genes up-regulated in each phenotype are also reported. To again ensure that there was not an effect of varied sample sizes among the six species in the proportion of DE genes identified, we employed the resampling approach described above where for all species except *Ceratina spp.*, N=3 samples per phenotype were resampled randomly without replacement, and DESeq was run again to find the DE genes. This was repeated 1000 times to enable us to calculate the number of times that the proportion of DE genes was more extreme than the point estimate. This number was then divided by 1000, resulting in a P-value that tests the null hypothesis that the point estimate is no different with all samples used if only N = 3 samples are used.

After ascertaining that there was no effect of uneven sample sizes in the identification of DE genes, the DE genes for each species were then aggregated and analyzed with TopGO for overlapping functions. The purpose of this was to search for a possible common ‘toolkit’ of gene functions rather than gene identity.

#### Machine Learning Analyses with Support Vector Machine (SVM)

Support Vector Machine (SVM) [(Cortes and Vapnik 1995)](https://paperpile.com/c/zTstmx/yX5Xx) is a supervised classification algorithm that can be used to predict phenotypes on the basis of data classification such as morphological measurements or expression patterns. It has been used to distinguish subtle differences in human cancer subtypes [(Yuan, Lu, and Zou 2020)](https://paperpile.com/c/zTstmx/rvmpS), to find non-expressed yet cancer-associated genes [(Ghanat Bari et al. 2017)](https://paperpile.com/c/zTstmx/mCO9C) and to explore microbiome [(Dhungel et al. 2021)](https://paperpile.com/c/zTstmx/ubQyT); as well as to contrast behavioral phenotypes in social Damaraland mole-rats [(Johnston et al. 2021)](https://paperpile.com/c/zTstmx/JAqTV), honey bees [(Liang et al. 2014)](https://paperpile.com/c/zTstmx/nPGWM) and the paper wasp *P. dominula* [(Taylor et al. 2021)](https://paperpile.com/c/zTstmx/qyHLc).

SVM is a complementary approach to conventional differential gene expression analysis such as DESeq2, and has proven successful classification accuracy in a benchmark study [(Zararsız et al. 2017)](https://paperpile.com/c/zTstmx/g13ur). This may make it a suitable choice for distinguishing subtle differences in gene expression that may be less likely to be detected by conventional DE methods due to either low sample sizes or noisier expression patterns, as seen in plastic phenotypes [(Taylor et al. 2021)](https://paperpile.com/c/zTstmx/qyHLc) or *in-silico* pooled data containing both septic and non-septic patient samples [(Schaack, Weigand, and Uhle 2021)](https://paperpile.com/c/zTstmx/4x8g8).

We used a train/test split approach, in which we test the data of each species against a model that has been trained on the other five species’ datasets. The result is a list of the 3,718 nearly single-copy orthologous genes predicted by the SVM as differentially expressed between reproductive (coded as *1*) and non-reproductive (coded as *0*) phenotypes.

In short, the raw read counts of the 3,718 orthologous genes in reproductive and non-reproductive samples from the six species (82 samples in total) were first transformed by variance stabilization in the DESeq2 R package [(Love, Anders, and Huber 2014; R Core Team 2014)](https://paperpile.com/c/zTstmx/skuKV+d8Ezg). To identify the appropriate kernel function, we calculated accuracy rates from SVM models run on each dataset using the e1071 R package [(Meyer et al. 2015)](https://paperpile.com/c/zTstmx/T4w1G) with linear and radial kernels and the following parameters: formula = phenotype ~ read counts, type = C-classification. Radial kernel consistently led to better prediction accuracy (i.e. higher accuracy rate for radial kernel than linear kernel). Thus, all subsequent models were fit using this kernel and a grid search of gamma between 10^-7^ and 10^-5^ and cost between 2^3^ and 2^5^. Next, for each of the six species, we constructed a full model with a k = 3 fold cross validation, in which a random third of the samples for that species is tested against the remaining all species’ samples as training data. K-fold Cross-Validation technique was recently benchmarked the best for sample size range of 20-100 [(Vabalas et al. 2019)](https://paperpile.com/c/zTstmx/bgnO2). We thus obtained a full-model prediction error rate for each species based on the predicted phenotype vs. the actual phenotype. Error rate is the performance measure of the prediction, specifically the mean squared error rate for regression (from the e1071 R package tune function): the smaller the error rate is, the better the SVM predicts the phenotype based on the read counts.

For feature selection (i.e. filtering for the genes that best predict the phenotype), we performed a leave-one-species-out iterative process, in which one species was chosen as the test dataset and the remaining five were used as the training set. Over 20 iterations, SVM models were run while fine-tuning the parameters of gamma and cost. The resulting model with the lowest error rate was used to assign weights to each of the 3,718 genes, where a higher weight meant that the gene was better at predicting the reproductive phenotype (coded as *1*). Feature weights were calculated by taking the matrix product of the coefficients for the model with its support vectors. We then performed recursive feature elimination, where at each step the gene with the lowest weight was removed, and the resulting model was again tuned using the remaining genes. This remove-one-gene cycle was iterated until the input dataset contained 100 genes only, and we selected the optimized model with the lowest error rate amongst these 3,618 best-performing models. Lastly, a final SVM model was run using the parameters and the gene predictors of the optimized model. This resulted in a set of gene predictors that represent DE genes between the reproductive and non-reproductive phenotypes for each of the six species. We then further filtered these predictor genes for those that overlap (1) across all six species and (2) overlap within just the bees or just the wasps.

#### Weighted Gene Co-expression Network Analysis (WGCNA)

As with the DE analysis, we took a two-prong approach to the construction of coexpression networks for species, both dependent on orthologs across species as well as independent of orthologs to facilitate assessment of shared gene function rather than gene identity. First, we constructed a multispecies coexpression (consensus) network to ask whether a conserved network exists and if modules within that network are associated with caste. These networks were orthology-dependent. Second, we constructed individual species’ networks that used all genes expressed in that species (i.e., orthology-independent). This second approach allowed us to ask whether there were any conserved functions of caste-biased expression missed by the first analysis.

##### Multispecies Consensus WGCNA

Using a method analogous to the ortholog-dependent DE analysis, we performed a consensus WGCNA [(Langfelder and Horvath 2008)](https://paperpile.com/c/zTstmx/I2J5) on the variance-stabilized read counts of the 3,718 nearly single-copy orthologs across all six species of bees and wasps. We repeated these analyses on the 5,787 and 6,983 nearly single-copy orthologs in bees and wasps, respectively. After removing genes with zero expression or low variance across samples, N = 1,507, N = 2,712, and N = 2,986 orthologs went into each of the analyses for the bees + wasps, bees, and wasps, respectively (Supplementary Table 13).

For each of the three consensus networks, soft-thresholding powers were identified for each species using an iterative process. For each species, we chose the lowest power for which the scale free topology fit R^2^ exceeded 0.8. These soft-thresholding powers were then used with a manual construction method that, for each species, was used to calculate the correlation adjacency matrix up to the soft-thresholding power for that species. These adjacency matrices were then used to calculate the Topological Overlap Matrix (TOM) for each species, which contains the topological similarity between genes based on coexpression. After scaling the TOMs for each species, a consensus TOM was found by taking the component-wise minimum of the TOMs across the species. The dissimilarity of this consensus TOM (*1* – *TOM*) was then used as the input to identify modules based on hierarchical clustering. Average linkage hierarchical clustering uses a dynamic tree cutting algorithm to cluster genes with high interconnectedness together into coexpression modules.

These modules represent network structure and organization but are dependent on the parameter passed for the minimum module size. Thus, we employed a novel, iterative approach to the minimum module size parameter of the hierarchical clustering step. This parameter sets the smallest number of genes required for a valid module during clustering. Because a smaller percentage of our orthogroups have sufficient read counts across all species to be informative in WGCNA, we reduced the minimum module size parameter iteratively. We report the results for minimum module size of 10 and 30 (Supplementary Table 13).

We then performed a meta-analysis on these module eigengenes (hereafter, ‘modules’) by calculating a transformed Z-score [(Langfelder et al. 2011)](https://paperpile.com/c/zTstmx/uxn7) to test whether modules are correlated with our focal traits of reproductive vs. non-reproductive phenotype across our six species. A similar method was used to test whether individual orthologs are correlated with modules and with traits. From there, orthologs that were significantly correlated with the trait-associated modules and correlated with either reproductive- or non-reproductive status were used for functional analyses. We consider these the significantly trait-associated genes conserved across the six species of bees and wasps. We then repeated all above steps for bees and wasps separately to generate lists of conserved clade-specific trait-associated genes.

We further tested whether the orthologs that were significantly correlated with the trait-associated modules and reproductive- or non-reproductive status deviated from a null expectation. If these orthologs were due to random chance, we expect that if we reshuffle the reproductive status labels and perform consensus WGCNA again, we will find the original set of genes 50% of the time because there are two traits (reproductive, non-reproductive). In brief, we shuffled the reproductive status labels before performing network construction. We then proceeded with the correlation meta-analyses on the coexpression networks as described above. We did this reshuffling *k* = 1,000 times and compared the significant orthologs obtained from each resampling event to the original list (i.e., the point estimate). We then calculated the proportion of point estimated genes found in a given resampling event. We derived a two-tailed p-value as the number of times this proportion was 50% or greater divided by the number of *k* resampling events. We did this for all three consensus networks: bees + wasps, bees only, and wasps only (Supplemental Table 13).

##### Orthology-Independent (Individual Species) Coexpression Networks

For each species individually, we constructed a weighted gene co-expression network on all reads after first applying the variance-stabilizing function on raw read counts in DESeq2 [(Love, Anders, and Huber 2014)](https://paperpile.com/c/zTstmx/skuKV) and filtering for the default minimum expression level employed by the WGCNA package. For individual species’ network construction, all genes were included that met the minimum expression level regardless of shared evolutionary history. After constructing the network on all genes, we then tested for correlation with our phenotypes of interest, reproductive vs. non-reproductive. This allowed us to ask whether there were any conserved functions of phenotype-biased expression missed by the consensus analysis. Genes from modules significantly associated with alternative social phenotypes were used to test for functional enrichment.

#### Functional Interpretation of Candidate Genes

We performed Gene Ontology Term enrichment analysis for genes identified by each of the three methods as related to our alternative social phenotypes, either genes differentially expressed (DESeq2), predictor genes (SVM), or genes coexpressed in modules associated with phenotype (WGCNA). To test for function enrichment of genes, we obtained the best similarity hits against the Drosophila melanogaster protein set using BLASTp V.2.2.30 [(Altschul et al. 1990)](https://paperpile.com/c/zTstmx/5Sqsr) for each species’ protein set. We then obtained enriched GO Terms using R biomaRt v. 2.42.1 [(Durinck et al. 2005)](https://paperpile.com/c/zTstmx/61noU) and TopGO v. 2.38.1 [(Alexa and Rahnenfuhrer 2010)](https://paperpile.com/c/zTstmx/0WGE3) with the following parameters: terms with at least 5 annotated genes, classic algorithm, and Fisher statistics. We also performed a GO Slim Terms enrichment analysis by obtaining D. melanogaster GO Slim Terms from GeneOntology website [(Ashburner et al. 2000; Gene Ontology Consortium 2021)](https://paperpile.com/c/zTstmx/TPgHG+KaVGr) and using Galaxy GOSlimmer Version 1.0.1 [(Afgan et al. 2016)](https://paperpile.com/c/zTstmx/Y8m3U) to obtain GO Slim Terms for the genes that had a top BLAST hit for a given wasp or bee species. We then used TopGO for the enrichment analysis of DEGs and WGCNA genes (i.e. found in modules significantly associated with reproductive status).

We also performed a REVIGO analysis of the enriched GO terms from 127 SVM predictor genes (parameters: medium resulting list, species: *Drosophila melanogaster*, SimRel measure [(Supek et al. 2011)](https://paperpile.com/c/zTstmx/HQe8O)), reducing the GO Term list to less redundant Terms. Finally, we queried the 127 SVM predictor genes against curated lists of Transcription Factors (TF, FlyBase version FB2021_03 [(Larkin et al. 2021)](https://paperpile.com/c/zTstmx/Fmcam) and FlyMine [(Lyne et al. 2007)](https://paperpile.com/c/zTstmx/YFcD6)) to test for over-representation of TF in the SVM predictor genes.

We further performed a REVIGO analysis of the shared GO terms from 71 genes in common to SVM and WGCNA.

## **Results**

#### Transcriptomes are of similar quality and completeness for downstream analyses.

Although the number of samples per RNAseq experiment per species ranged from 6 to 24, we find that the quality and completeness of the mapped RNAseq reads are comparable across the six species (Supplementary Table 5). The proportion of uniquely mapped reads ranged from 64.5% to 95.6%, with the lowest proportion of uniquely mapping reads in *C. calcarata* and the highest in *P. canadensis.* Generally, the proportions are similar across all species except the two *Polistes* spp. where the proportions of uniquely mapped reads were above 90%.

We find that the level of BUSCO completeness of longest-isoform protein sets (between 65% and 88%, Supplementary Table 3) is in the lower end of established Hymenoptera UniProt protein sets (around 90%), which might reduce our capacity to obtain a complete set of true single-copy orthologs common to all six species. Using a relaxed filtering approach in which we allow between one and three gene copies per species and for an orthogroup to be absent in up to one species, we find 3,718 near single-copy orthogroups common to all six species, 5,787 common to the three bees and 6,983 common to the three wasps (Supplementary Table 6). Notably, we do not find any apparent expression bias in the 3,718 orthogroups for any of the species (Supplementary Figure 2). This, alongside no outlier in plots (PCA Figure 1, read count heatmap Supplementary Figure 18, ROC Supplementary Figure 20), shows that the difference of tissue type (all brains except for whole-head *C. calcarata*) does not have an impact on further analyses.

#### Minimal commonly differentially expressed (DE) genes between alternative social phenotypes using conventional analyses

We took a two-pronged analytical approach where we asked whether there are common differentially-expressed (DE) genes across the six species of bees and wasps or, if due to deep evolutionary divergence, common functions of DE genes. To ask the former question, we took an orthology-dependent approach, which compared orthologous DE genes across bees + wasps, as well as bees and wasps independently. We then looked for overlapping DE genes as well as gene functions. For the latter approach, we did species-specific DE analyses (Supplementary Figure 2), aggregated DE genes, and then looked for common functional annotations across those DE genes pooled across the six species.

The proportion of all genes of the six species with at least one read in at least one sample ranged from 64.2% in *C.australensis* to 98.3% in *P. dominula* (mean 89.4%, Supplementary Table 5). Because of this variation, proportions of differentially expressed (DE) genes are reported rather than counts. Point estimates for the proportion of all genes DE expressed between reproductive and non-reproductive phenotypes were calculated using all reads with at least one read (hereafter the ‘transcriptome’) as the denominator. The sizes of the transcriptomes varied from 10,214 (*P. canadensis*) to 14,606 (*C. australensis*) (Supplementary Table 5).

##### Orthology-dependent approach reveals no commonly differentially expressed genes between alternative social phenotypes either across bees and wasps or within each clade

We find that the proportion of orthologs with some expression (at least one read in at least one sample) ranged from 80.6% in *C. calcarata* to 97.1% in *L. flavolineata* (mean 89.5%, Supplementary Table 18A). We obtained point estimates for the proportion of orthologs differentially expressed between primary reproductive and non-reproductive phenotypes. We found that these varied across the six species, with point estimates ranging from 0.35% (*P. canadensis*) to 13.3% (*P. dominula*) (Supplementary Table 18A). Because sample sizes per species varied, we used a resampling approach to test if the point estimate of proportion of DE orthologs differed if the number of samples per species and per phenotype was restricted to *N = 3.*

Notably, we only find an effect of a restricted sample size of *N* = 3 for *P. dominula* (P << 0.001, Supplementary Table 18A) but not *M. genalis*, *P. canadensis,* or *L. flavolineata* (Supplementary Figure 3). Neither *Ceratina* spp. were resampled because only *N* = 3 samples were available. *P. dominula* also had the largest number of available samples (*N =* 12) per phenotype, which may suggest that the larger number of samples in *P. dominula* allowed for identification of a higher proportion of DE orthologs in that species. We know that the resampling approach is quite robust because any given DE ortholog from the full dataset was seen upon resampling anywhere from 87.6% (*P. dominula*) to 100% (*P. canadensis*) of the time (Supplementary Table 18A). This suggests that only for *P. dominula* is there a possible effect of sample size on our ability to detect common DE orthologs among the species. On average, most DE orthologs are seen 3-4 times per 1000 iterations per species (Supplementary Table 18A). However, given that resampling produced highly variable estimates of the proportion of DE orthologs for *P. dominula* (Supplementary Table 18A, Supplementary Figure 3) it is unlikely that this reflects a true difference in the proportion of DE genes in *P. dominula*.

Thus, we conclude that similar proportions of DE orthologs between social phenotypes exist across bees and wasps. However, the identities of the DE orthologs do not overlap between the lineages (Supplementary Table 19A). No single orthogroup was differentially expressed in common across all six species; the most seen was across four species. However, these were not seen across the same species each time, and a limiting factor with this analysis was the few DEGs identified in *P. canadensis.* Further, GO analysis revealed no common functional enrichment across these genes at the P < 0.05 level. Importantly, when looking across the bees and wasps individually, we also find very minimal commonality (Supplementary Tables 19B-C). For example, there is only one orthogroup commonly differentially expressed across the three bees, and there were no orthogroups commonly differentially expressed across the three wasps.

##### Orthology-independent approach reveals no shared functions of differentially expressed genes between alternative social phenotypes either across bees and wasps

The proportions of genes differentially expressed between primary reproductive and non-reproductive phenotypes varied across the six species, with point estimates ranging from 0.29% (*P. canadensis*) to 11.83% (*P. dominula*) (Supplementary Table 18B). Because sample sizes per phenotype varied, a resampling approach was used to test whether (1) the point estimate of proportion of DEGs in the transcriptome differed if the number of samples per phenotype were restricted to N = 3 and (2) to test whether the proportion of DEGs in the transcriptome differs among lineages. We only find an effect of a restricted sample size of N = 3 for *P. dominula* (P << 0.001, Supplementary Table 18B) but not *M. genalis*, *P. canadensis*, or *L. flavolineata* (Supplementary Figure 4). Neither *Ceratina spp*. were resampled because only N = 3 samples were available. *P. dominula* also had the largest number of available samples (N = 12) per phenotype, which may suggest that the larger number of samples in *P. dominula* likely allowed for identification of a higher proportion of DEGs in that species. Thus, for all species except *P. dominula*, a reduced sample size has no effect on the proportion of DE genes identified in the transcriptome. Further, resampling is able to identify a given DE gene at least 86.5% of the time (*P. dominula*) and up to 100% of the time (*P. canadensis*) with resampling Supplementary Table 18B). On average, most DE genes are seen 3-4 times per 1000 iterations.

Thus, we conclude that similar proportions of DE genes between social phenotypes exist across bees and wasps regardless of shared evolutionary history among those genes. To test whether there is a common function across those DE genes, functional annotation of the aggregate DE genes from the six species were performed with TopGO. The aggregate DE genes mapped to 938 gene ontology categories. Of these GO categories, none were significantly enriched at the FDR-adjusted P < 0.05 level, and none were represented across more than two of the species (Supplementary Table 20).

#### Support Vector Machine analysis finds a small number of genes with conserved caste-biased expression between bees and wasps, as well as within clade.

Using a leave-one-species-out approach, we tested each species (number of samples between six and 24) against a training set of the other five species (number of samples between 57 and 75) with an SVM classifier of reproductive vs. non-reproductive phenotype using gene expression for the 3,718 nearly single-copy orthogroups as input. The number of predictor genes identified between reproductive and non-reproductive phenotypes ranged from 10.7% to 17.1% of orthogroups across the six species (Supplementary Table 7A, Supplementary Figures 5-10). With the exception of *P. canadensis*, recursive selection resulted in a lower error rate than when all samples from all species were used to predict DE genes (Supplementary Table 7A, Supplementary Figure 22). Recursive feature selection resulted in a mean error rate of 18.9% (SD 2.7%). This means that the optimized models with tuned parameters and predictor genes misclassified reproductive phenotypes across all six species 18.9% of the time on average.

We find 127 predictor genes common to all six species (Figure 4a). SVM rank (*i.e.* how important an orthogroup predicts the phenotype) does not cluster species according to lineage (Supplementary Figure 17), nor do the variance-stabilized read counts (Supplementary Table 7, Supplementary Figure 18). Thus, the significance of those 127 predictor genes is likely the result of small effects from many genes in each species, due to life history differences between species.

The analysis was repeated for the bee and wasp lineages as well to see if there were lineage specific predictor genes that might shed light on common genes or functions due to shared life history (Figure 1). Again using a leave-one-species-out approach, we tested each species against a training set of the other two species within the lineage for bees and wasps, respectively. We again used the SVM classifier of reproductive vs. non-reproductive phenotype on gene expression data for the nearly single-copy orthogroups as input (N = 5,787 bees; N = 6,983 wasps). The number of predictor genes identified between reproductive and non-reproductive phenotypes ranged from 8.7% to 18.9% of orthogroups for bees, and from 10.2% to 25.9% for wasps (Supplementary Table 7B, Supplementary Figures 11-16). For all species, recursive selection resulted in a lower error rate than when all samples from all species were used to predict DE genes (Supplementary Table 7B). Recursive feature selection resulted in a mean error rate of 28.0% (SD 0.9%) for bees and 17.0% (SD 0.2%) for wasps. This means that the optimized models with tuned parameters and predictor genes misclassified reproductive phenotypes across all the three bee or wasp species 28% and 17%, respectively, of the time on average. The higher error rate for bees versus wasps may reflect the lower sample sizes (N = 3) for the two *Ceratina* spp. For the bees, 56 common predictor genes were identified (Supplementary Table 10) representing genes with functions like sex differentiation, although none of the functions were significantly enriched at the P < 0.05 level. For the wasps, 148 common predictor genes were identified (Supplementary Table 11) representing genes with functions like head development or chromatin organization, although none of the functions were significantly enriched at the P < 0.05 level.

#### Consensus WGCNA identifies a different, but overlapping, set of caste-biased genes from SVM

We also asked whether patterns of coexpressed genes might be conserved across our six species of bees and wasps (Supplementary Table 12A) or within each clade (Supplementary Tables 12B and 12C, respectively). Using a novel, iterative approach where we relax the minimum module size (to allow for smaller modules and thus smaller clusters of coexpression), we find that there is more coexpression with more species. The consensus network with all six species of bees and wasps is a larger network than those with just bees or just wasps (Supplementary Table 12). Thus, we focus primarily on the results of the multispecies consensus network for all six species but report the brief results for each lineage as well (Supplementary Table 13).

Using a meta-analysis to identify (*Z* summary score) modules significantly associated with alternative social phenotype across bees + wasps, we find that there are 1 to 12 modules, depending on the relaxation of the minimum module size parameter (Figure 4 and Supplementary Table 13A). Of the 568 genes in the largest network (minimum module size parameter = 30), 137 genes were independently associated with reproductive and non-reproductive phenotype in a separate meta-analysis (Supplementary Table 13A). We also find that this is unlikely to result from random chance after performing a resampling test (P << 0.001). We hypothesized that if significant trait-associated orthologs in consensus modules were a result of random chance, we would see a high proportion (at or near 50%) of point estimated genes upon reshuffling of the reproductive status labels.

For bees only, not surprisingly we find a much larger consensus network that ranges from 13 to 33 modules, depending on the relaxation of the minimum module size parameter (Supplementary Table 13B). Of the 2,343 genes in the largest network (minimum module size parameter = 30), 591 genes were independently associated with reproductive and non-reproductive phenotype in a separate meta-analysis (Supplementary Table 13B). We again find that this is unlikely to result from random chance after performing a resampling test (P = 0.002).

For wasps only, the consensus network is also much larger. It ranges from 9 to 18 modules, depending on the relaxation of the minimum module size parameter (Supplementary Table 13C). Of the 3,885 genes in the largest network (minimum module size parameter = 30), 832 genes were independently associated with reproductive and non-reproductive phenotype in a separate meta-analysis (Supplementary Table 13C). We further find that this is unlikely to result from random chance after performing a resampling test in which we reshuffled the reproductive status labels before calling the consensus network (P << 0.001).

Overall, of the orthogroups identified by the meta-analysis that were significantly trait-associated with minimum module size = 30, N = 27 (21.3%) of these overlap with the 127 predictor genes common to bees + wasps from the SVM analysis, were identified in the consensus trait-associated module. When the more relaxed approach with a minimum module size of 10 was used, allowing for smaller, tighter modules, N = 71 of the 127 SVM genes overlapped (55.9%, Supplementary Table 17). We also have confidence in the significantly trait-associated orthologs in consensus modules, as they were robust when compared to null expectations (Supplementary Table 13A-C).

#### Individual species’ coexpression networks reveal no shared patterns of gene functional enrichment

We constructed WGCNA networks for each species individually regardless of gene homology (orthology-independent). This allowed us to ask whether there were any conserved functions of phenotype-biased expression missed by the consensus analysis. Using WGCNA and all genes with sufficient expression for network construction for each species, we constructed species-specific networks. Not surprisingly, we found different numbers of modules of varying sizes for each species (Supplementary Table 15). The number of modules that were significantly associated with social phenotype ranged from 2-5 across the six species. There was also variation in the numbers of genes that were in these significantly trait-associated modules, ranging from 290 to 4,290. The *Polistes* spp. had the fewest genes in phenotype-associated modules. These phenotype-associated genes were tested for functional enrichment with TopGo (Supplementary Table 16**).** Although there were no functions that overlapped across all six species, there were common enriched functional terms across three to four species, including those involved in ribonucleotide synthesis, oxidoreductase processes, and ATP metabolism (Supplementary Table 16**).**

#### Transcription factor analyses

We queried the 127 SVM predictor genes against curated lists of Transcription Factors (TF, FlyBase version FB2021_03 [(Larkin et al. 2021)](https://paperpile.com/c/zTstmx/Fmcam) and FlyMine [(Lyne et al. 2007)](https://paperpile.com/c/zTstmx/YFcD6)) to test for over-representation of TF in the SVM predictor genes. We find a significant under-representation of Transcription Factors (TF) in the predictor genes (one-sided Fisher’s exact test on number of TFs in non-SVM orthogroups and in SVM orthogroups, p = 0.03388).

#### Semantic similarity in GO Terms enriched in all methods (SVM, DEGs, WGCNA)

Finally, we also extended our exploration by qualifying the genes that are common to the SVM, WGCNA and differentially-expressed analyses. We looked for semantic similarity in enriched GO Terms overlapping between the differentially expressed genes, the WGCNA genes and the SVM predictor genes (Supplementary Figure 18, Supplementary Table 9). We found several terms such as immune system process, regulation of developmental process, negative regulation of chromatin organisation and sensory functions such as olfactory and chemosensory behaviour. Several of these terms have previously been associated with reproductive phenotypes in bee heads [(Vojvodic et al. 2015)](https://paperpile.com/c/zTstmx/fgTyl) and with the evolution of communication systems in social bees [(Wittwer et al. 2017)](https://paperpile.com/c/zTstmx/mIMKg).

##

## Figures


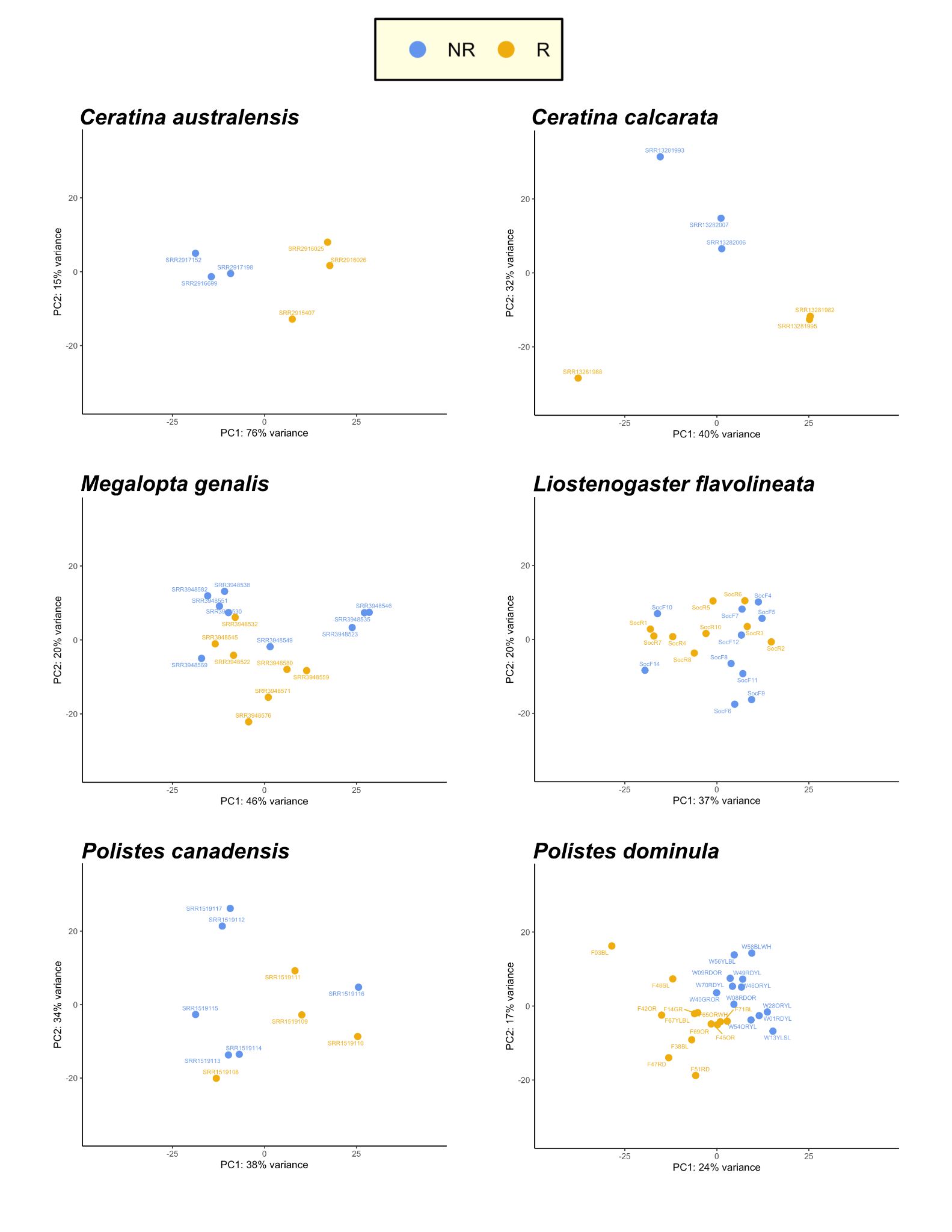


#### Supplementary Figure 1. Principal Component plots for the expression data showing Reproductive (R) vs. non-reproductive (NR) phenotypes for the 6 species in this study.

PC plots show that some species have more distinct expression patterns (e.g., *Ceratina australensis*) than other species (e.g., *Polistes canadensis*) between their alternative social phenotypes.


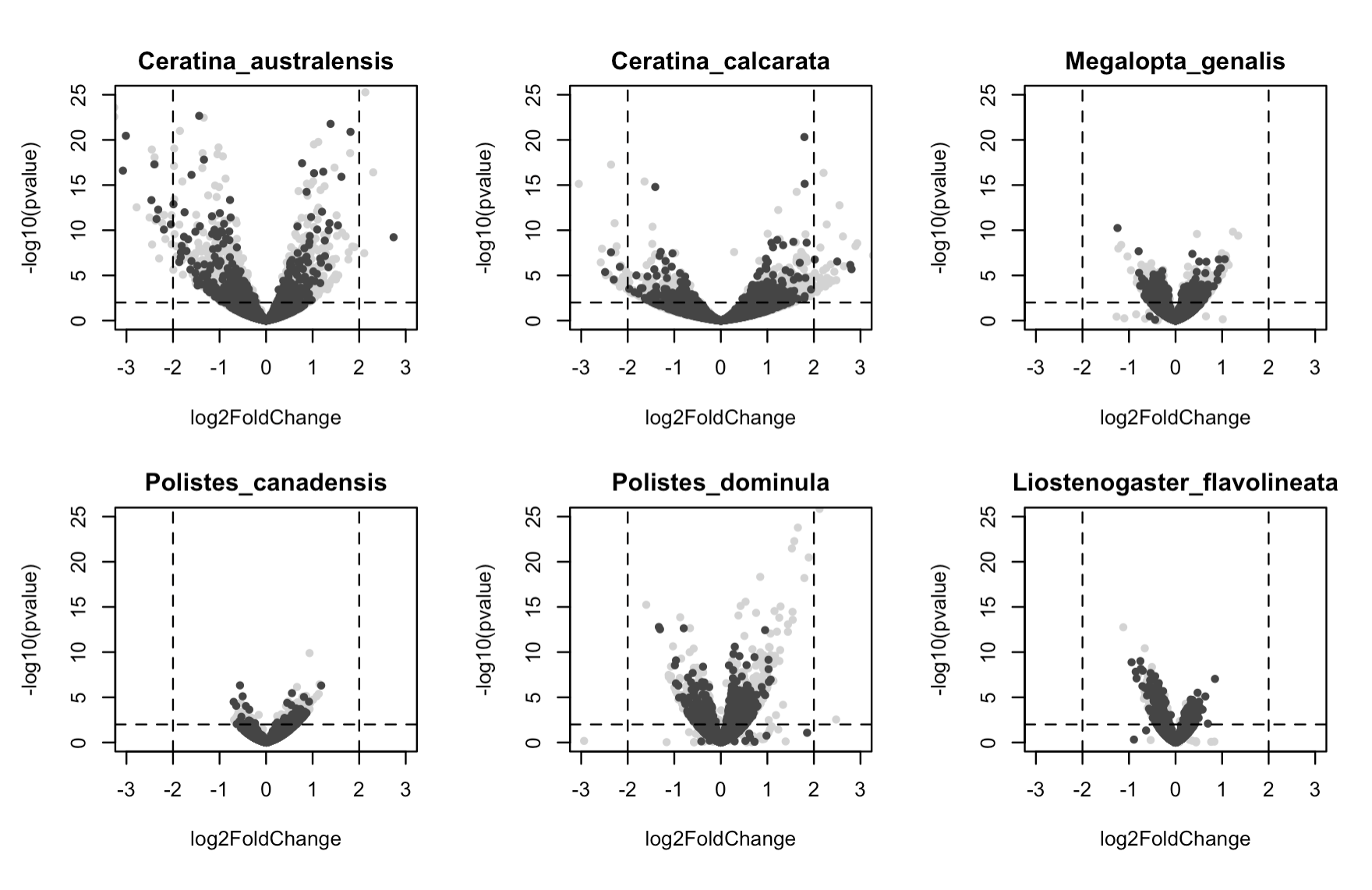


#### Supplementary Figure 2. No expression bias in the 3,718 near single-copy orthogroups common to the three bee and three wasp species used in this study.

Volcano plots show the expression pattern for all genes in the transcriptome (light gray) versus orthogroups (dark gray) for each of the six species. Volcano plots show the statistical significance (y-axis, -log_10_ scale; -log_10_(0.05) = 1.3) versus the magnitude of expression change (log_2_-fold change). Horizontal dashed lines demarcate a p-value of 0.05, and vertical dashed lines demarcate log_2_-fold change expression more extreme expression than two log-fold. These six species vary in their transcriptomic patterns, but all species show the pattern of most orthogroups have modest expression levels and a handful of genes with higher magnitude expression levels.


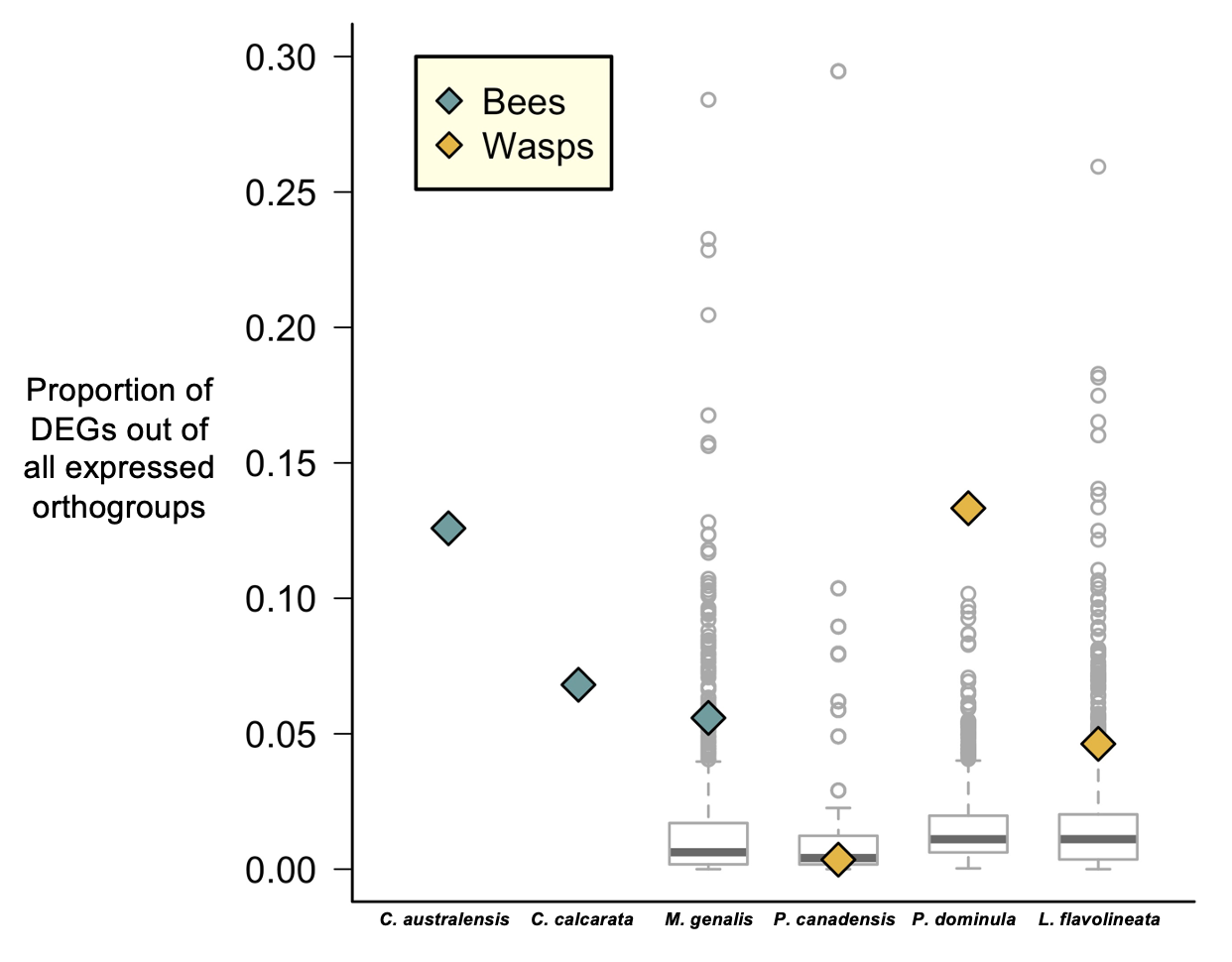


#### Supplementary Figure 3. Effect of restricted (N = 3) sample sizes in identification DE orthogroups in species with N > 3 RNAseq samples.

Shown are the proportions of all expressed orthogroups (N = 3,718, both bees and wasps) that were identified as differentially-expressed between alternative social phenotypes after resampling to test for any effect of sample size variation among the RNAseq studies analyzed. In short, the proportions of genes differentially expressed between primary reproductive and non-reproductive phenotypes were calculated with DESeq2. Because sample sizes per phenotype varied, a resampling approach was used to test whether (1) the point estimate of proportion of DEGs in the transcriptome differed if the number of samples per phenotype were restricted to N = 3 and (2) to test whether the proportion of DEGs in the transcriptome differs among lineages. We only find an effect of a restricted sample size of N = 3 for *P. dominula* (P << 0.001, Supplementary Table 18B) but not *M. genalis*, *P. canadensis*, or *L. flavolineata*. Neither *Ceratina spp*. were resampled because only N = 3 samples were available but their point estimates are shown for comparison to the other species.

**
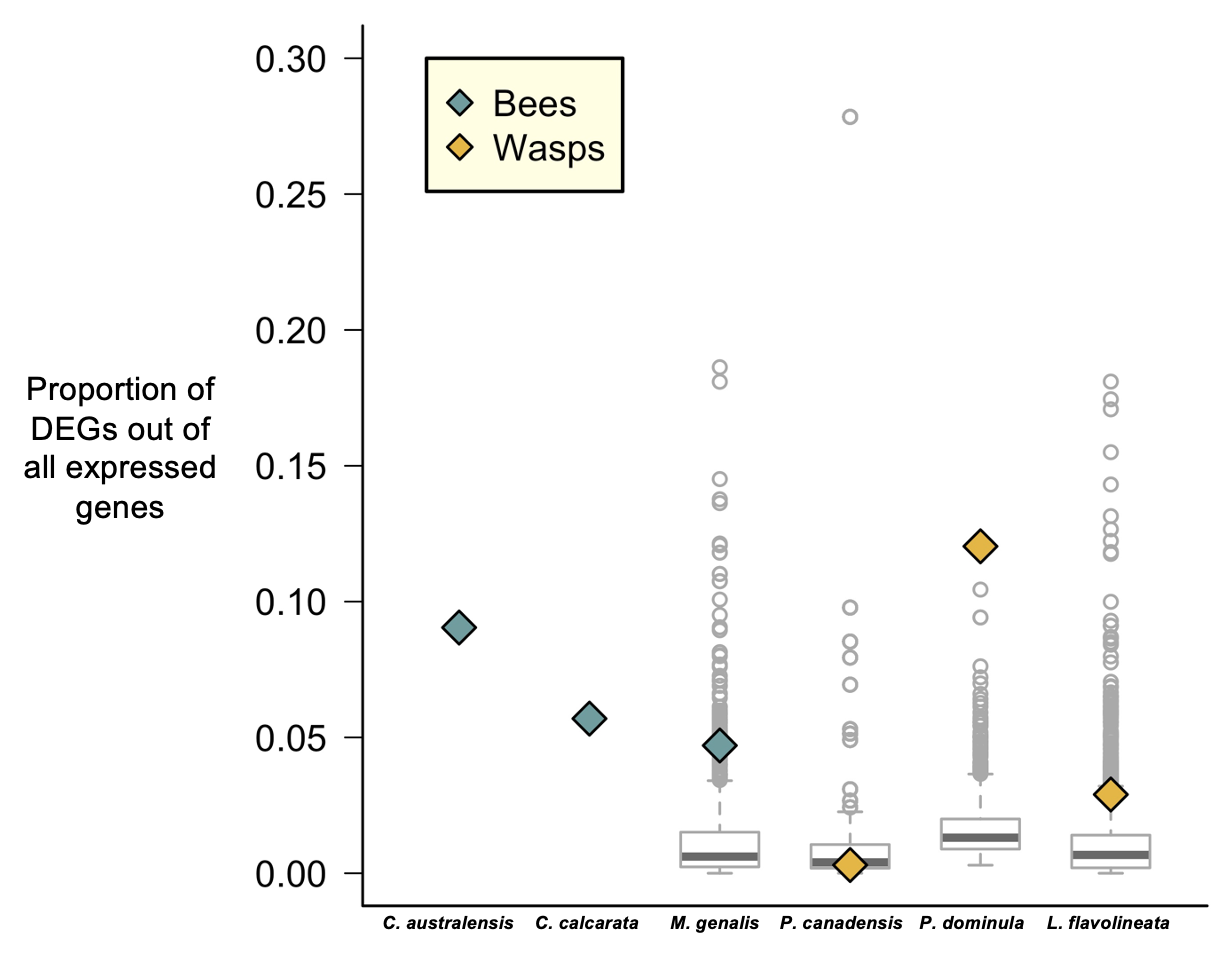
**

#### Supplementary Figure 4. Effect of restricted (N = 3) sample sizes in identification DE genes in species with N > 3 RNAseq samples.

Shown are the proportions of all expressed genes that were identified as differentially-expressed between alternative social phenotypes after resampling to test for any effect of sample size variation among the RNAseq studies analyzed. In short, the proportions of genes differentially expressed between primary reproductive and non-reproductive phenotypes were calculated with DESeq2. Because sample sizes per phenotype varied, a resampling approach was used to test whether (1) the point estimate of proportion of DEGs in the transcriptome differed if the number of samples per phenotype were restricted to N = 3 and (2) to test whether the proportion of DEGs in the transcriptome differs among lineages. We only find an effect of a restricted sample size of N = 3 for *P. dominula* (P << 0.001, Supplementary Table 18B) but not *M. genalis*, *P. canadensis*, or *L. flavolineata*. Neither *Ceratina spp*. were resampled because only N = 3 samples were available but their point estimates are shown for comparison to the other species.


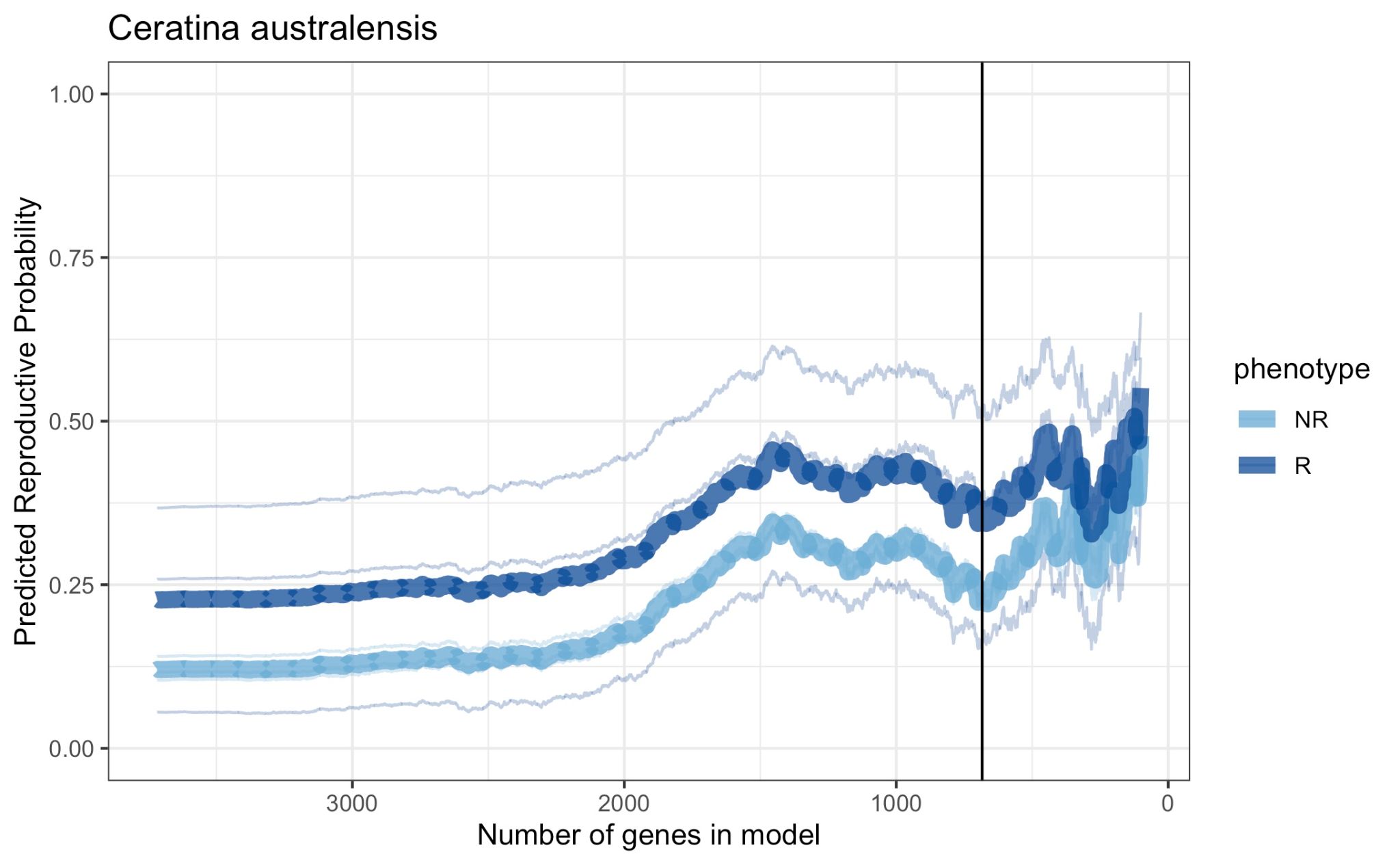


#### Supplementary Figure 5. SVM prediction of *Ceratina australensis* phenotype using a large training set.

Starting with 3,718 orthogroups, the recursive feature selection (x-axis) ran until 100 orthogroups were left in the model, which was trained with data from the five other species (2 bees, 3 wasps; total number of samples = 75). The phenotype of each sample (n = 6) in the focus species is predicted, with a probability of 1 for reproductive (R) and 0 for non-reproductive (NR). The best model with the lowest error rate is highlighted with the vertical black line, and includes 684 genes.


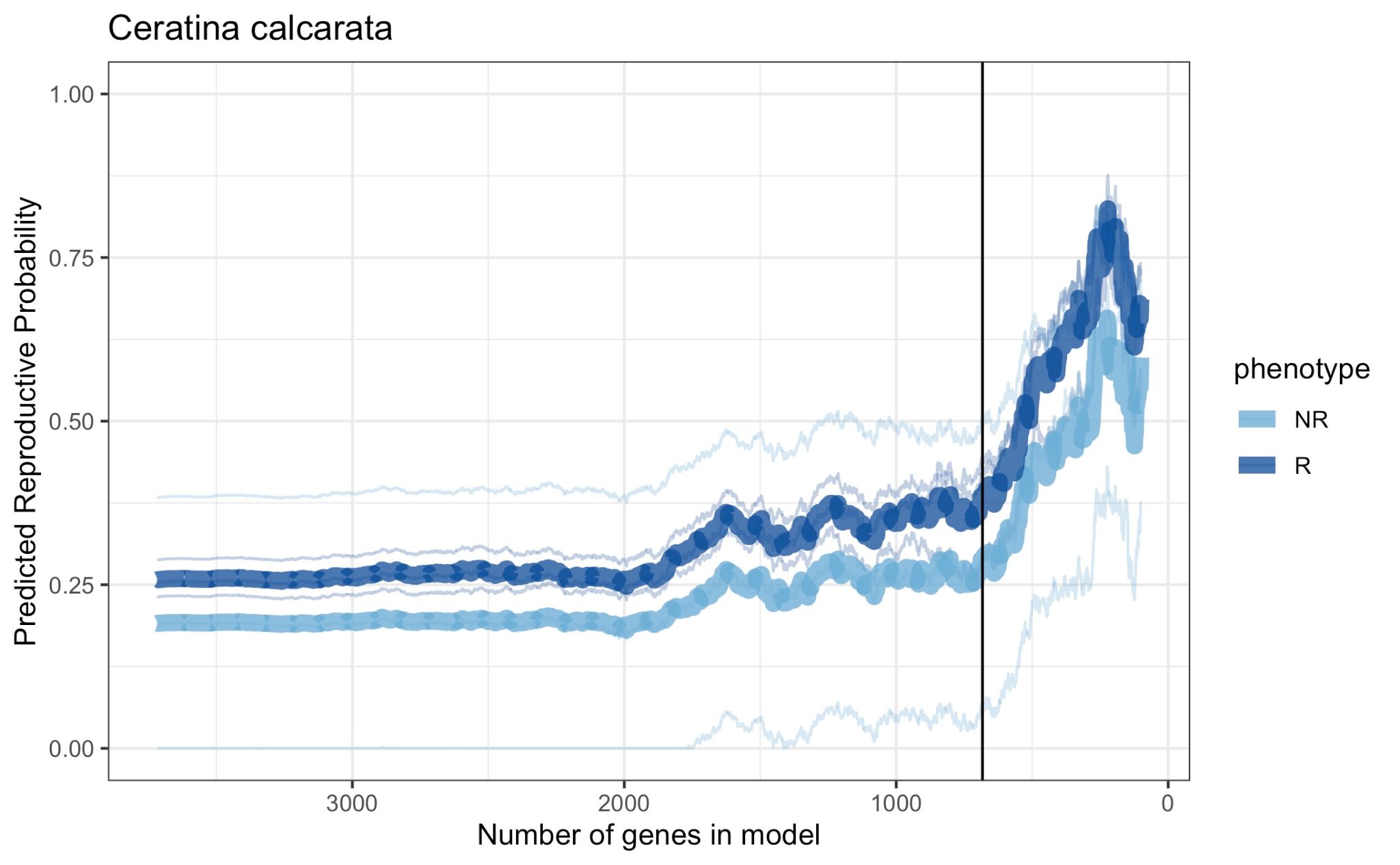


#### Supplementary Figure 6. SVM prediction of *Ceratina calcarata* using a large training set.

Starting with 3,718 orthogroups, the recursive feature selection (x-axis) ran until 100 orthogroups were left in the model, which was trained with data from the five other species (2 bees, 3 wasps; total number of samples = 75). The phenotype of each sample (n = 6) in the focus species is predicted, with a probability of 1 for reproductive (R) and 0 for non-reproductive (NR). The best model with the lowest error rate is highlighted with the vertical black line, and includes 683 genes.


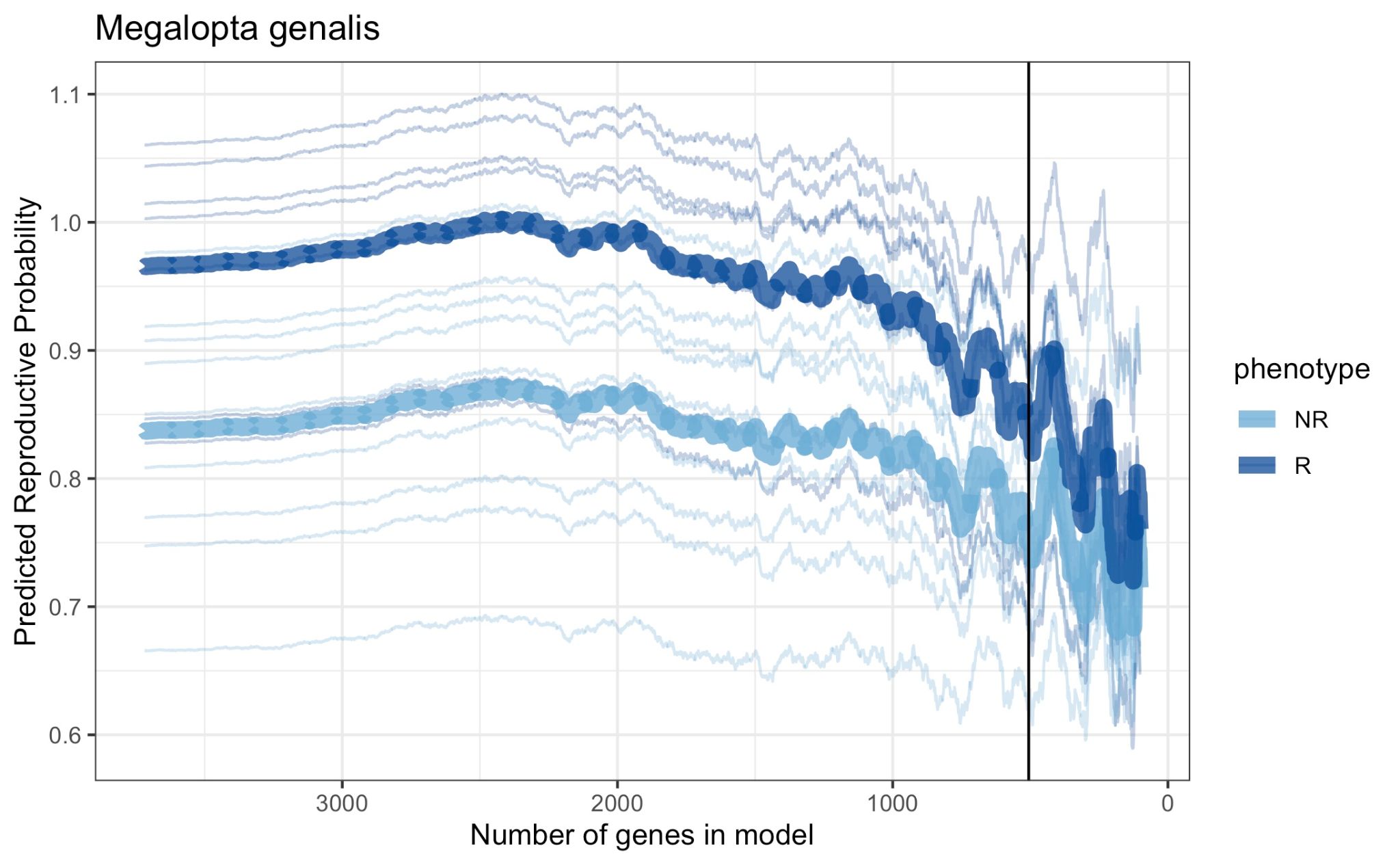


#### Supplementary Figure 7. SVM prediction of *Megalopta genalis* using a large training set.

Starting with 3,718 orthogroups, the recursive feature selection (x-axis) ran until 100 orthogroups were left in the model, which was trained with data from the five other species (2 bees, 3 wasps; total number of samples = 65). The phenotype of each sample (n = 16) in the focus species is predicted, with a probability of 1 for reproductive (R) and 0 for non-reproductive (NR). The best model with the lowest error rate is highlighted with the vertical black line, and includes 506 genes.


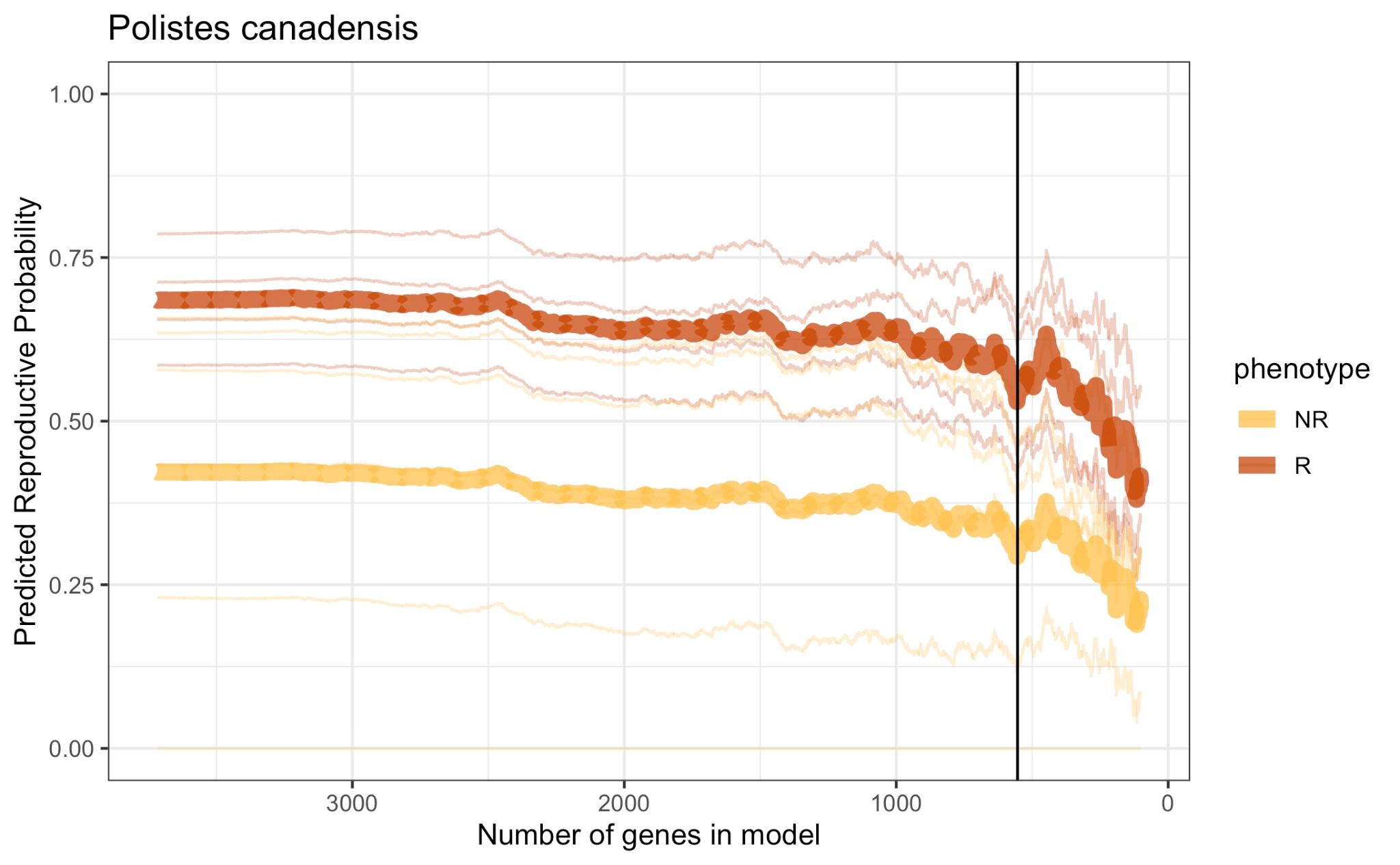


#### Supplementary Figure 8. SVM prediction of *Polistes canadensis* using a large training set.

Starting with 3,718 orthogroups, the recursive feature selection (x-axis) ran until 100 orthogroups were left in the model, which was trained with data from the five other species (3 bees, 2 wasps; total number of samples = 71). The phenotype of each sample (n = 10) in the focus species is predicted, with a probability of 1 for reproductive (R) and 0 for non-reproductive (NR). The best model with the lowest error rate is highlighted with the vertical black line, and includes 554 genes.


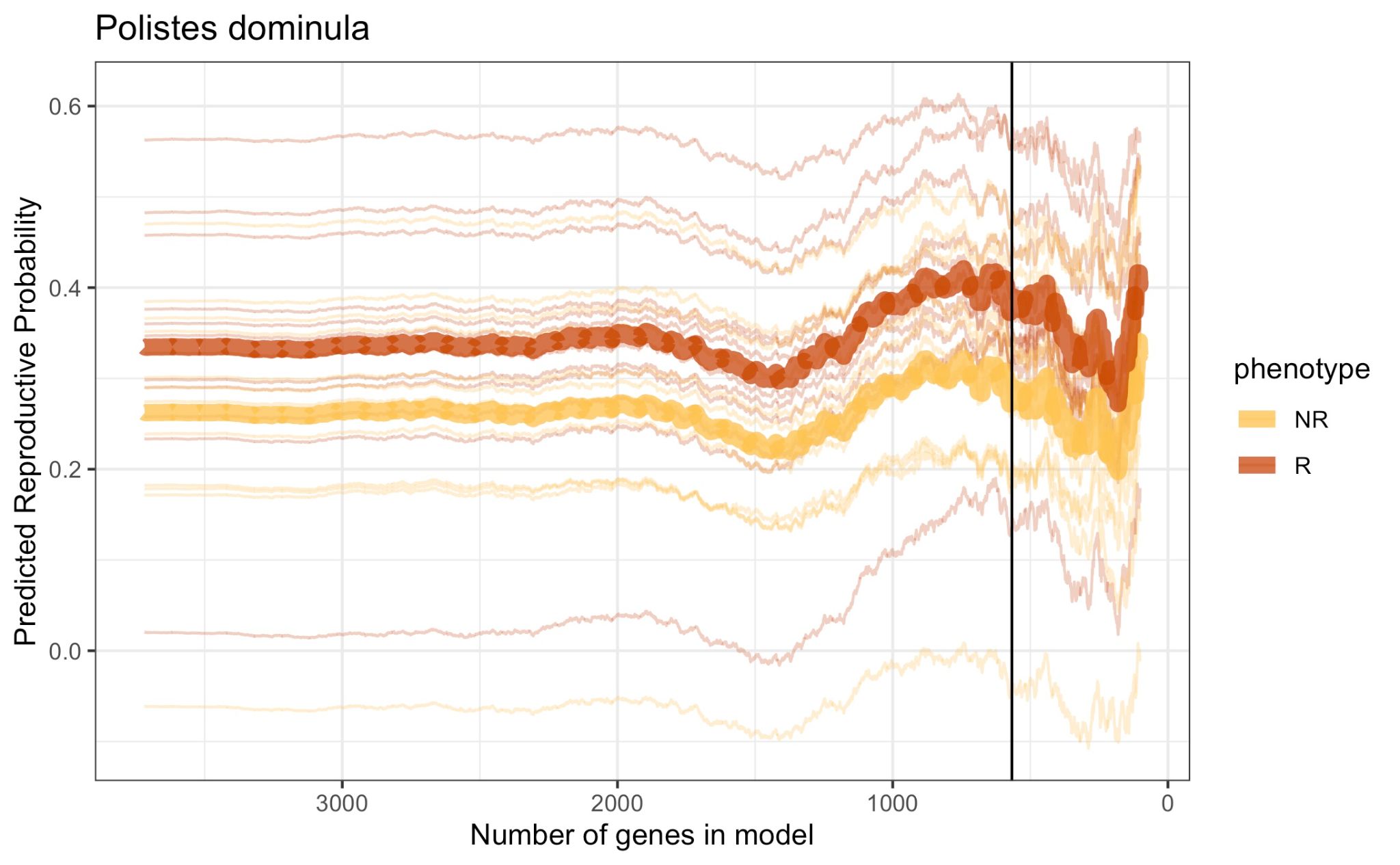


#### Supplementary Figure 9. SVM prediction of *Polistes dominula* using a large training set.

Starting with 3,718 orthogroups, the recursive feature selection (x-axis) ran until 100 orthogroups were left in the model, which was trained with data from the five other species (3 bees, 2 wasps; total number of samples = 57). The phenotype of each sample (n = 24) in the focus species is predicted, with a probability of 1 for reproductive (R) and 0 for non-reproductive (NR). The best model with the lowest error rate is highlighted with the vertical black line, and includes 567 genes.


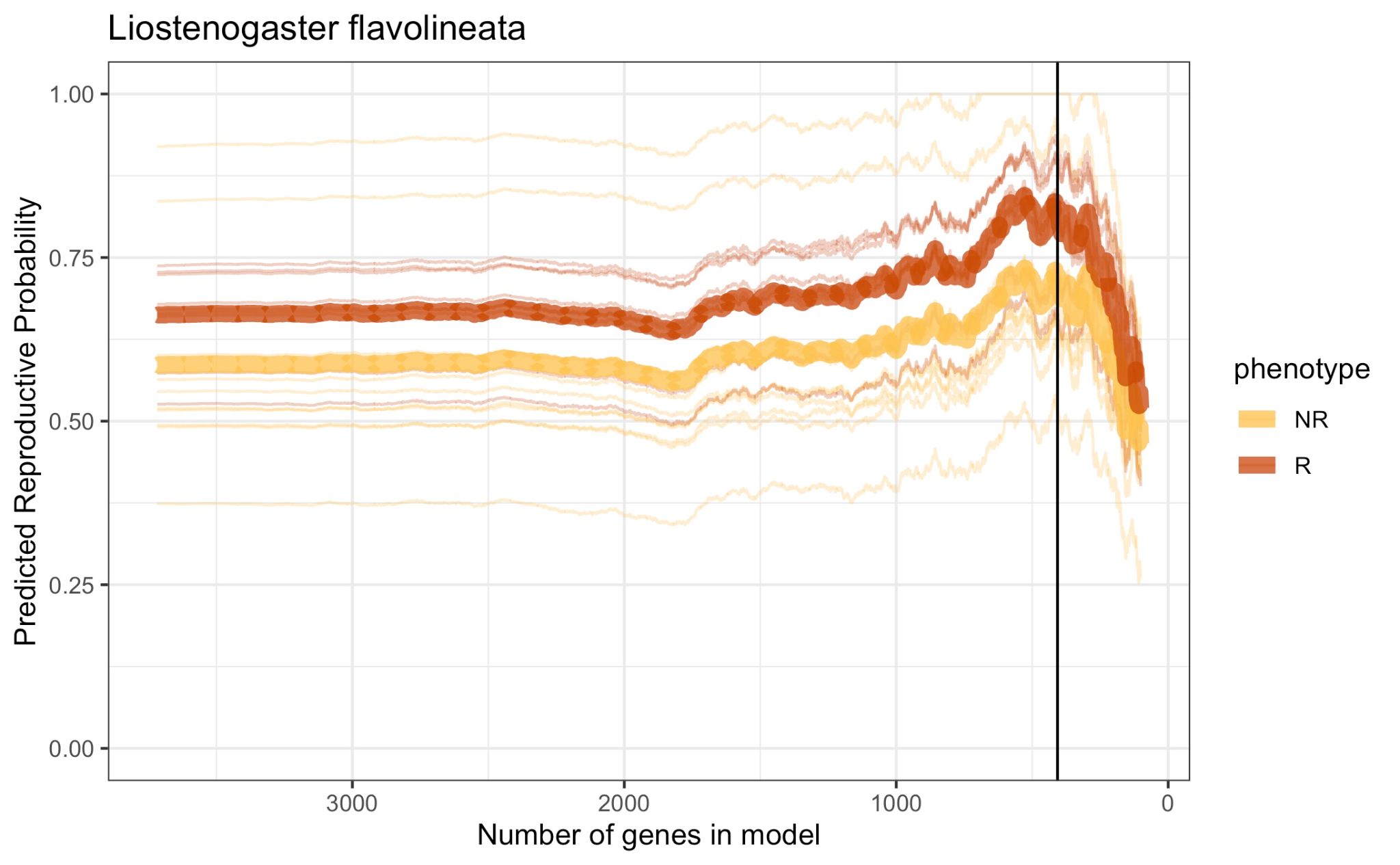


#### Supplementary Figure 10. SVM prediction of *Liostenogaster flavolineata* using a large training set.

Starting with 3,718 orthogroups, the recursive feature selection (x-axis) ran until 100 orthogroups were left in the model, which was trained with data from the five other species (3 bees, 2 wasps; total number of samples = 62). The phenotype of each sample (n = 19) in the focus species is predicted, with a probability of 1 for reproductive (R) and 0 for non-reproductive (NR). The best model with the lowest error rate is highlighted with the vertical black line, and includes 407 genes.


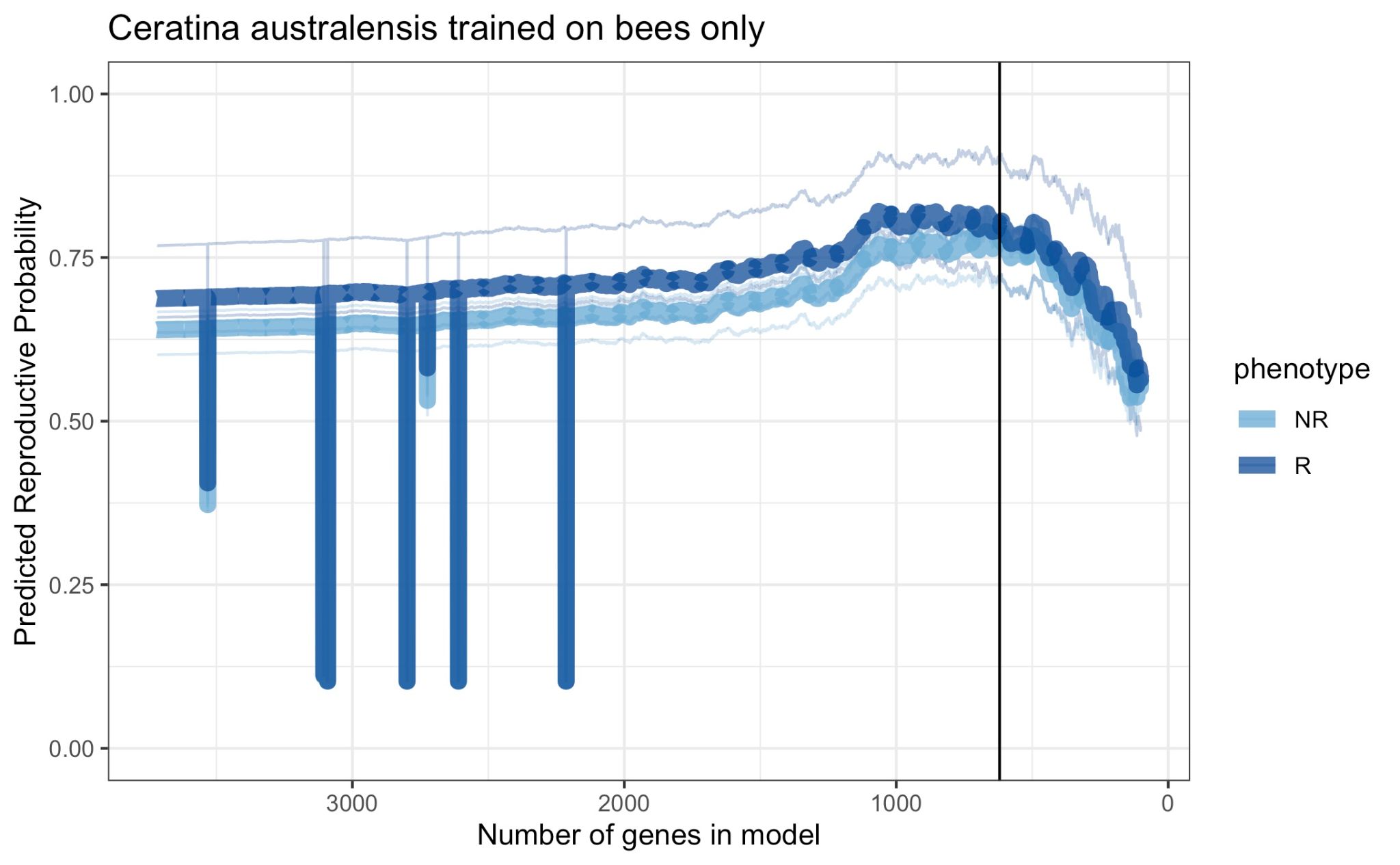


#### Supplementary Figure 11. SVM prediction of *Ceratina australensis* using a lineage-specific training set.

Starting with 3,718 orthogroups, the recursive feature selection (x-axis) ran until 100 orthogroups were left in the model, which was trained with data from the two other bee species (total number of samples = 22). The phenotype of each sample (n = 6) in the focus species is predicted, with a probability of 1 for reproductive (R) and 0 for non-reproductive (NR). The best model with the lowest error rate is highlighted with the vertical black line, and includes 476 genes.


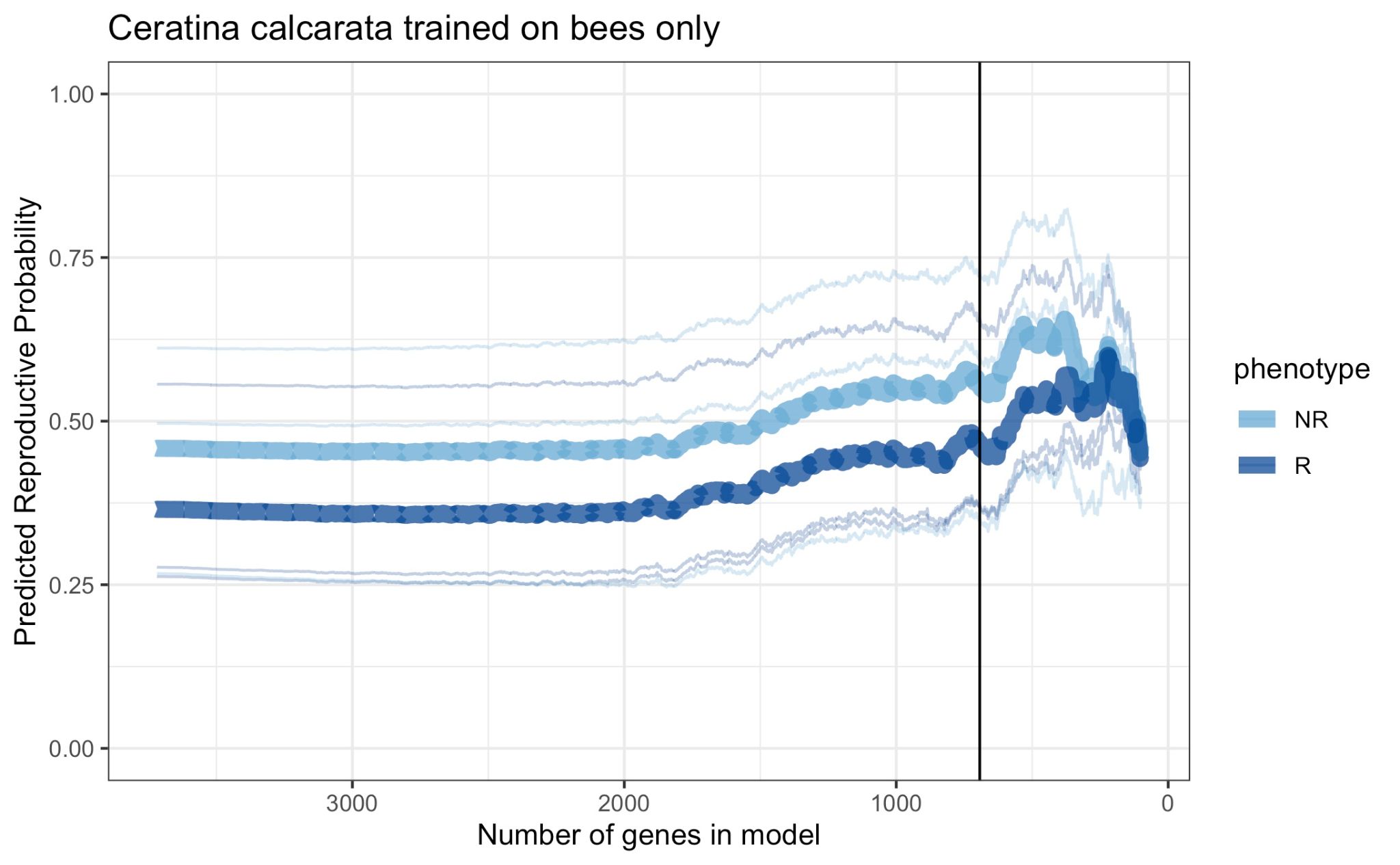


#### Supplementary Figure 12. SVM prediction of *Ceratina calcarata* using a lineage-specific training set.

Starting with 3,718 orthogroups, the recursive feature selection (x-axis) ran until 100 orthogroups were left in the model, which was trained with data from the two other bee species (total number of samples = 22). The phenotype of each sample (n = 6) in the focus species is predicted, with a probability of 1 for reproductive (R) and 0 for non-reproductive (NR). The best model with the lowest error rate is highlighted with the vertical black line, and includes 693 genes.


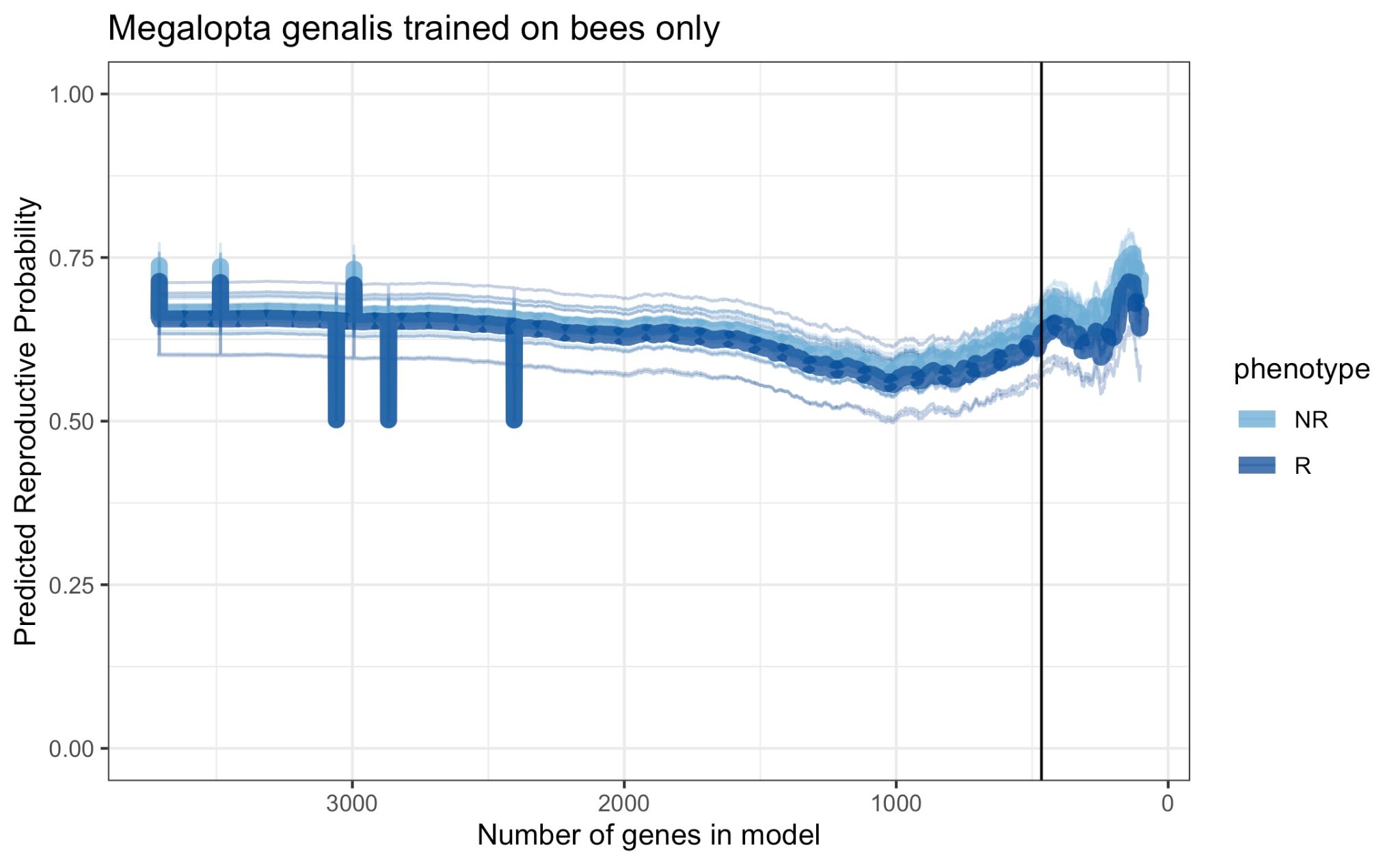


#### Supplementary Figure 13. SVM prediction of *Megalopta genalis* using a lineage-specific training set.

Starting with 3,718 orthogroups, the recursive feature selection (x-axis) ran until 100 orthogroups were left in the model, which was trained with data from the two other bee species (total number of samples = 12). The phenotype of each sample in the focus species (n = 16) is predicted, with a probability of 1 for reproductive (R) and 0 for non-reproductive (NR). The best model with the lowest error rate is highlighted with the vertical black line, and includes 466 genes.


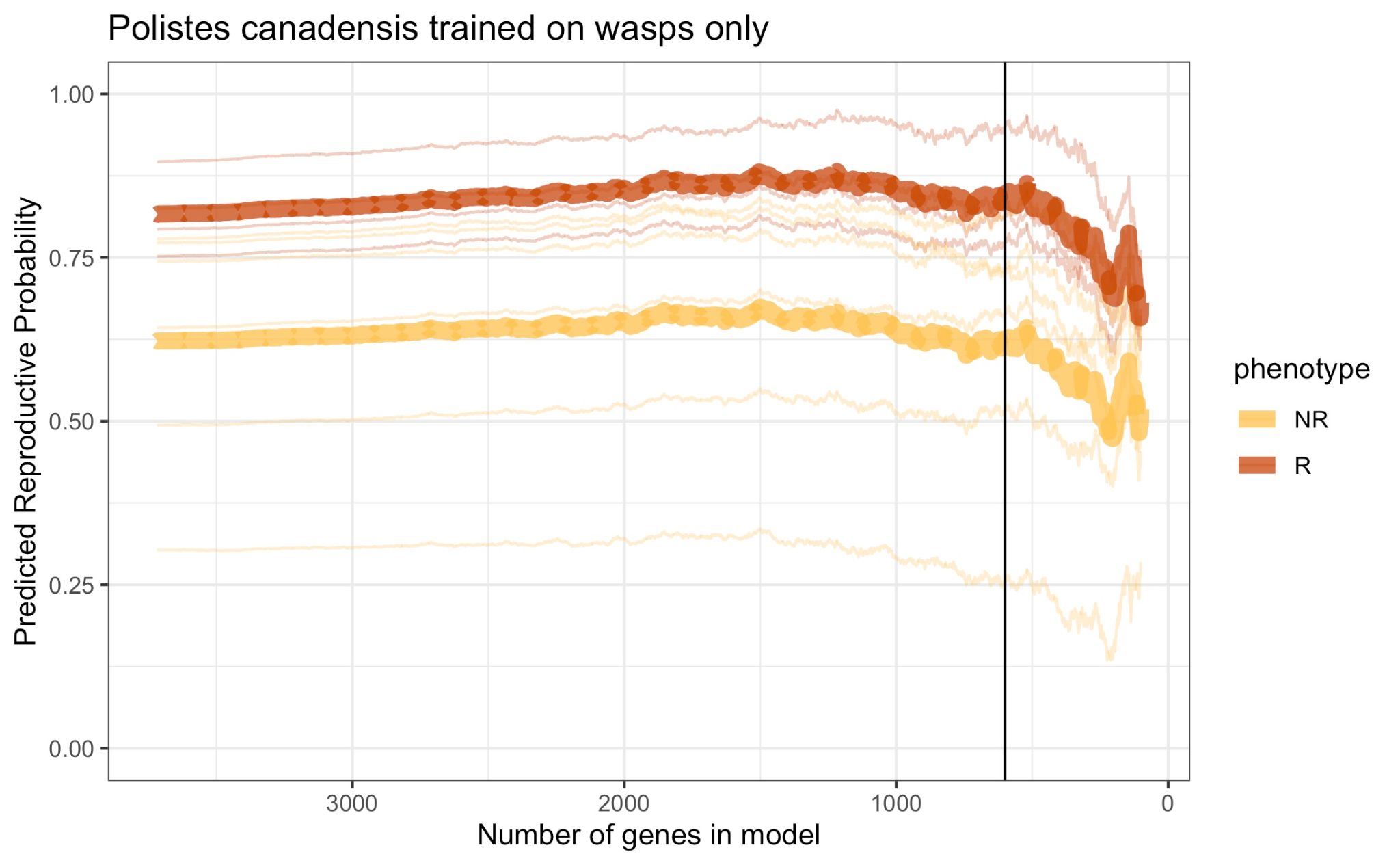


#### Supplementary Figure 14. SVM prediction of *Polistes canadensis* using a lineage-specific training set.

Starting with 3,718 orthogroups, the recursive feature selection (x-axis) ran until 100 orthogroups were left in the model, which was trained with data from the two other wasp species (total number of samples = 43). The phenotype of each sample in the focus species (n = 10) is predicted, with a probability of 1 for reproductive (R) and 0 for non-reproductive (NR). The best model with the lowest error rate is highlighted with the vertical black line, and includes 600 genes.


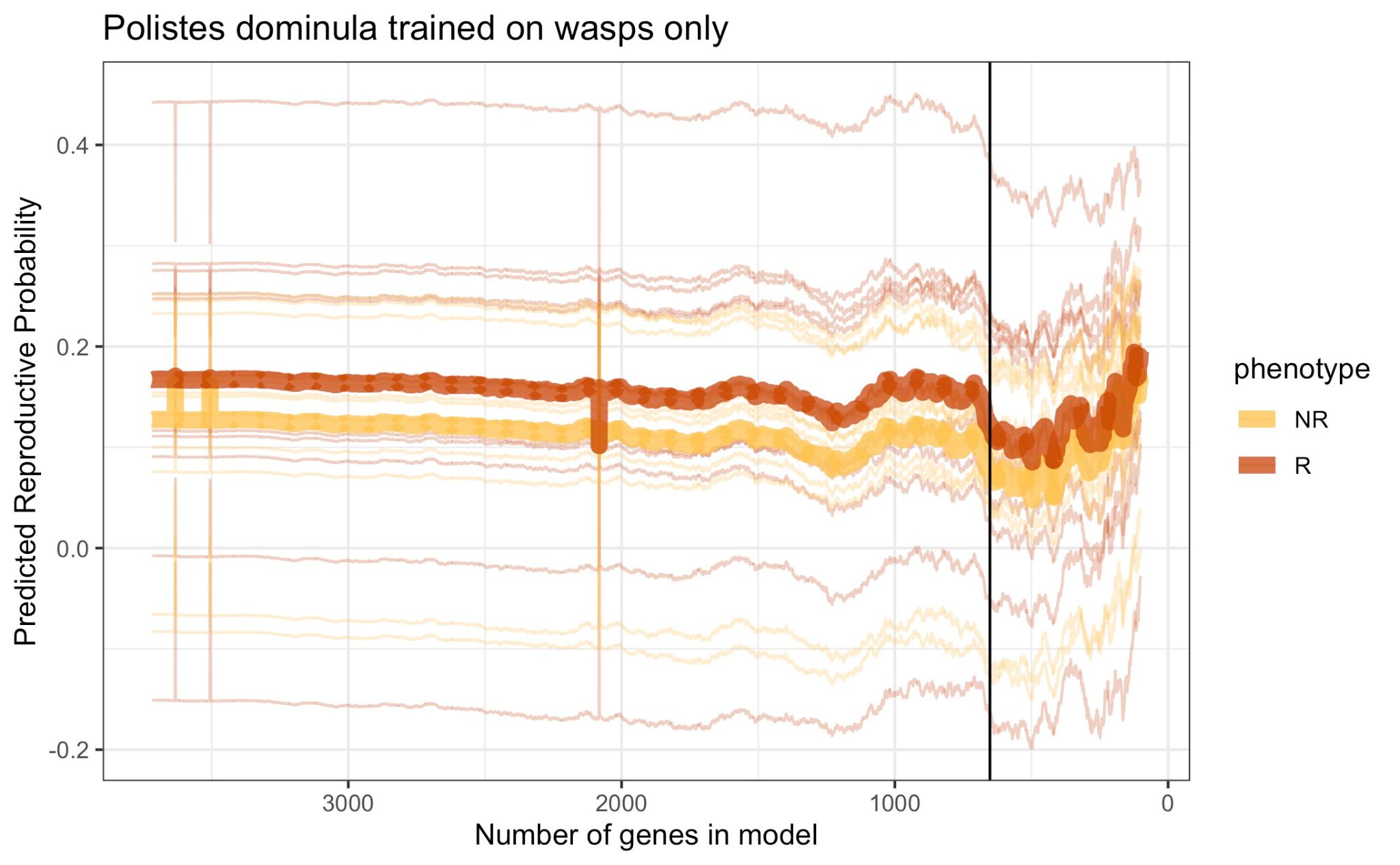


#### Supplementary Figure 15. SVM prediction of *Polistes dominula* using a lineage-specific training set.

Starting with 3,718 orthogroups, the recursive feature selection (x-axis) ran until 100 orthogroups were left in the model, which was trained with data from the two other wasp species (total number of samples = 29). The phenotype of each sample in the focus species (n = 24) is predicted, with a probability of 1 for reproductive (R) and 0 for non-reproductive (NR). The best model with the lowest error rate is highlighted with the vertical black line, and includes 652 genes.


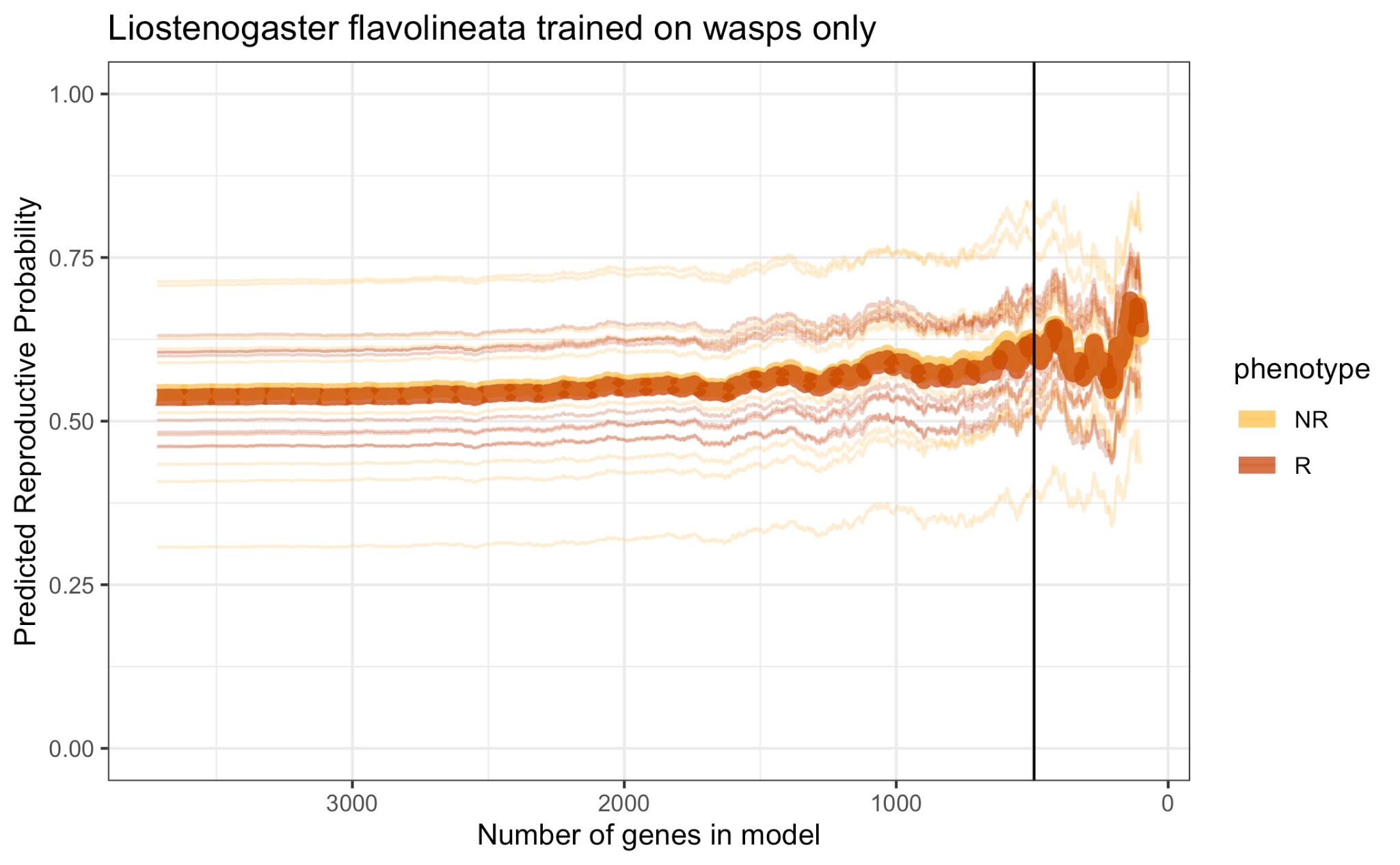


#### Supplementary Figure 16. SVM prediction of *Liostenogaster flavolineata* using a lineage-specific training set.

Starting with 3,718 orthogroups, the recursive feature selection (x-axis) ran until 100 orthogroups were left in the model, which was trained with data from the two other wasp species (total number of samples = 34). The phenotype of each sample in the focus species (n = 19) is predicted, with a probability of 1 for reproductive (R) and 0 for non-reproductive (NR). The best model with the lowest error rate is highlighted with the vertical black line, and includes 493 genes.

#### Supplementary Figure 17: Heatmap of 3,718 Orthologous Genes Shows that Overall Topology Differs from that Obtained by SVM Ranking.
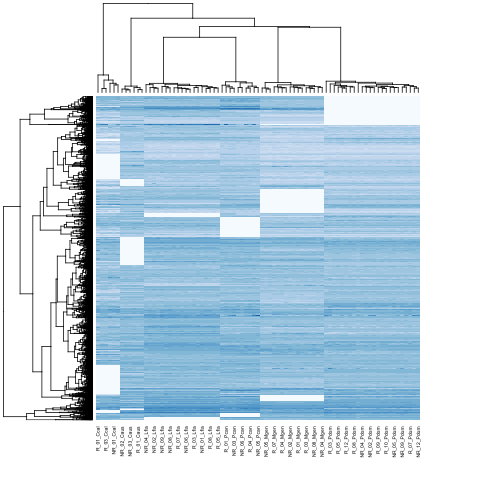


Read counts after variance stabilizing transformation (VST) do not cluster by taxonomy (e.g., *Polistes canadensis* is in a different clade than *Polistes dominula*). Phenotypes cluster together within species only for the *Ceratina* spp. Note that the overall topology differs from that obtained by SVM Ranking (Figure 3).


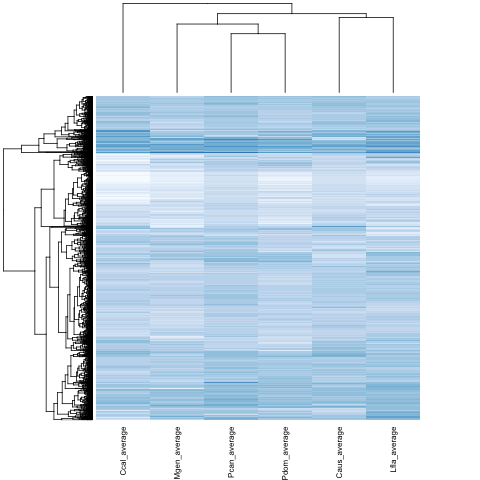


#### Supplementary Figure 18. Heatmap of 3,718 Orthologous Genes Shows that Overall Topology Differs from that Obtained by SVM Ranking.

VST read counts averaged by species do not cluster by taxonomy. Any orthogroup that had zero copy in a given species is removed from the analysis. The orthogroups are in row (n = 1583 orthogroups for which each species had some read counts). The species are in columns. The darker the colour, the more read counts. The hierarchical clustering ("complete" method) from the euclidean distance matrix of the species does not cluster taxa in a phylogenetic topology (i.e. wasp *Liostenogaster* clusters with bee *Ceratina australensis*).

#### Supplementary Figure 19. REVIGO semantic plot of GO terms common to SVM, WGCNA and DEG analyses.
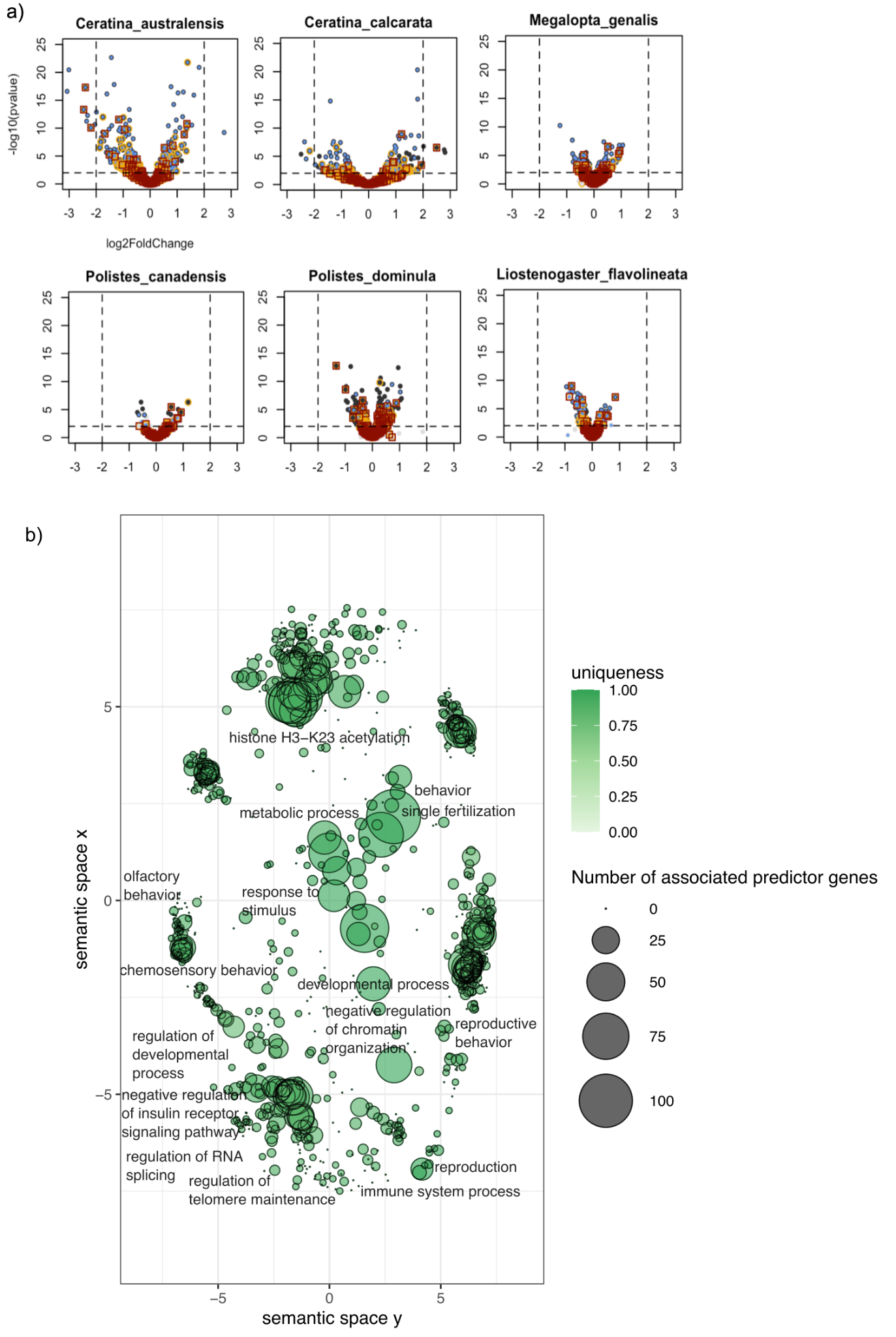


Revigo Semantic-similarity based multidimensional scaling of Biological Processes enriched GO Terms common to DEGs, WGCNA and SVM. The darker the point, the more unique the GO term is (REVIGO semantic algorithm). The larger the point, the more genes relate to the GO term.


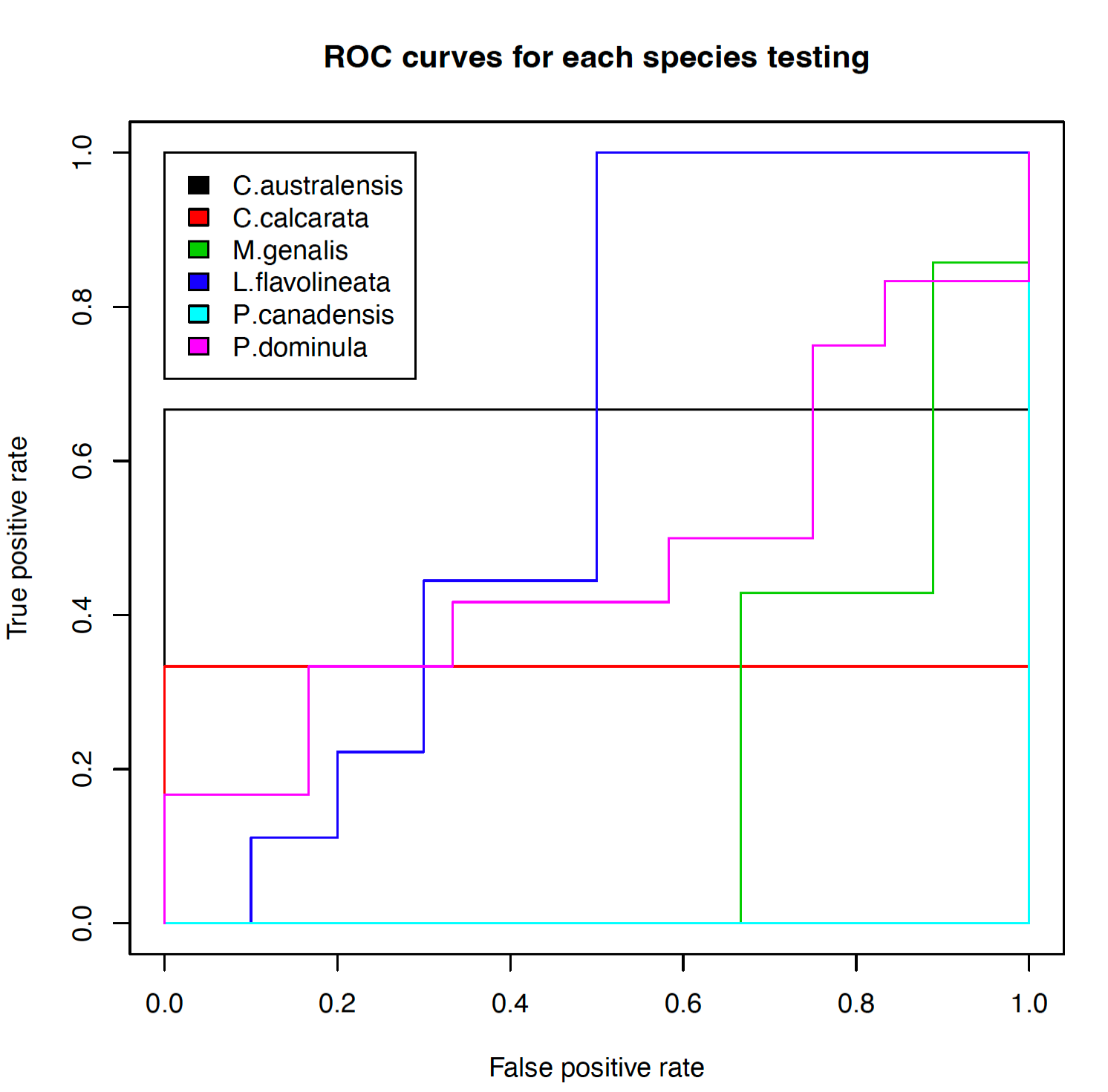


#### Supplementary Figure 20. Receiver Operating Characteristics curves of SVM models testing samples from one species against samples from the five other species.


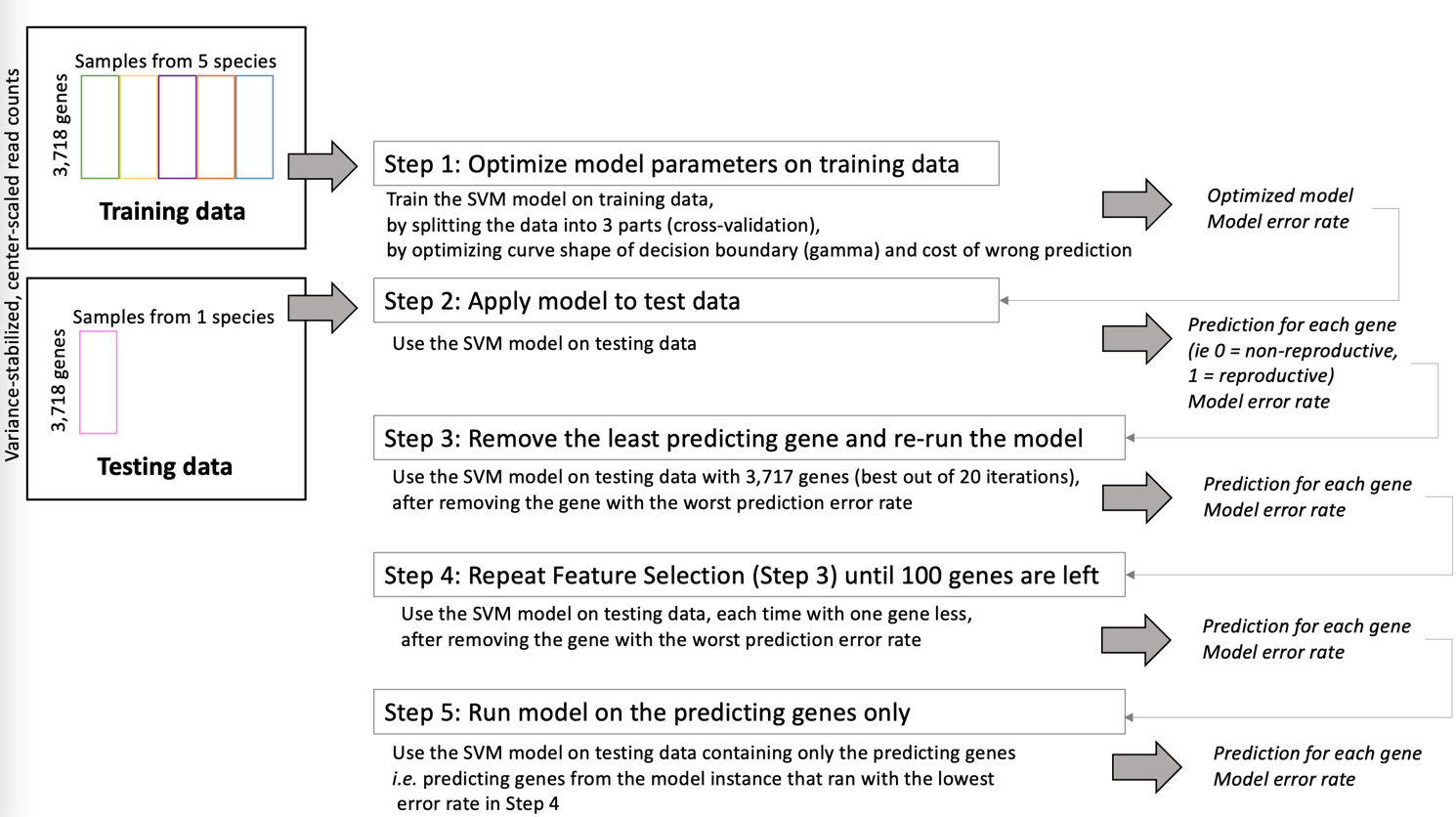


#### Supplementary Figure 21. Support Vector Machine and Feature Selection Model Flowchart

The input is normalized read counts of 3,718 genes in 82 samples of six species. For each species, the SVM is built using the other five species as training data (Step 1). The model is first applied to the test data as a whole (ie all genes; Step 2), and then to an ever decreasing number of genes (ie removing the worst predicting genes; Step 3 and 4). Eventually a subset of best predictor genes is selected when the SVN model error rate is the lowest (Step 5).

####
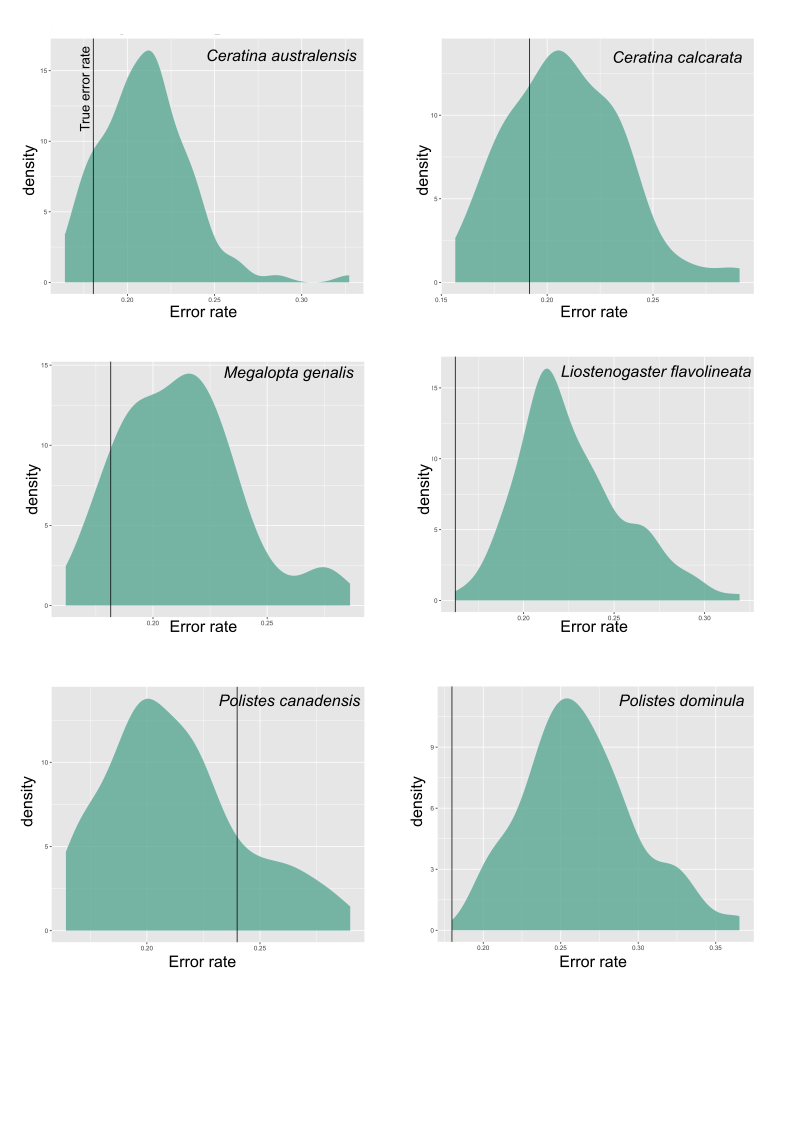
Supplementary Figure 22. SVM Randomization Tests

Samples from each focal species had their phenotype randomized prior to be tested by SVM over 100 iterations. SVM error rates (x axis: best prediction equal 0) are reported as a density curve (y axis: less counts toward 0), to be compared to the true error rate (vertical black line). The true error rate falls into the first decile rank for three species (*Ceratina australensis*, *Liostenogaster flavolineata*, *Polistes dominula*), into the second decile rank for two species (*C. calcarata,* *Megalopta genalis*), into the ninth decile rank for *P. canadensis*.


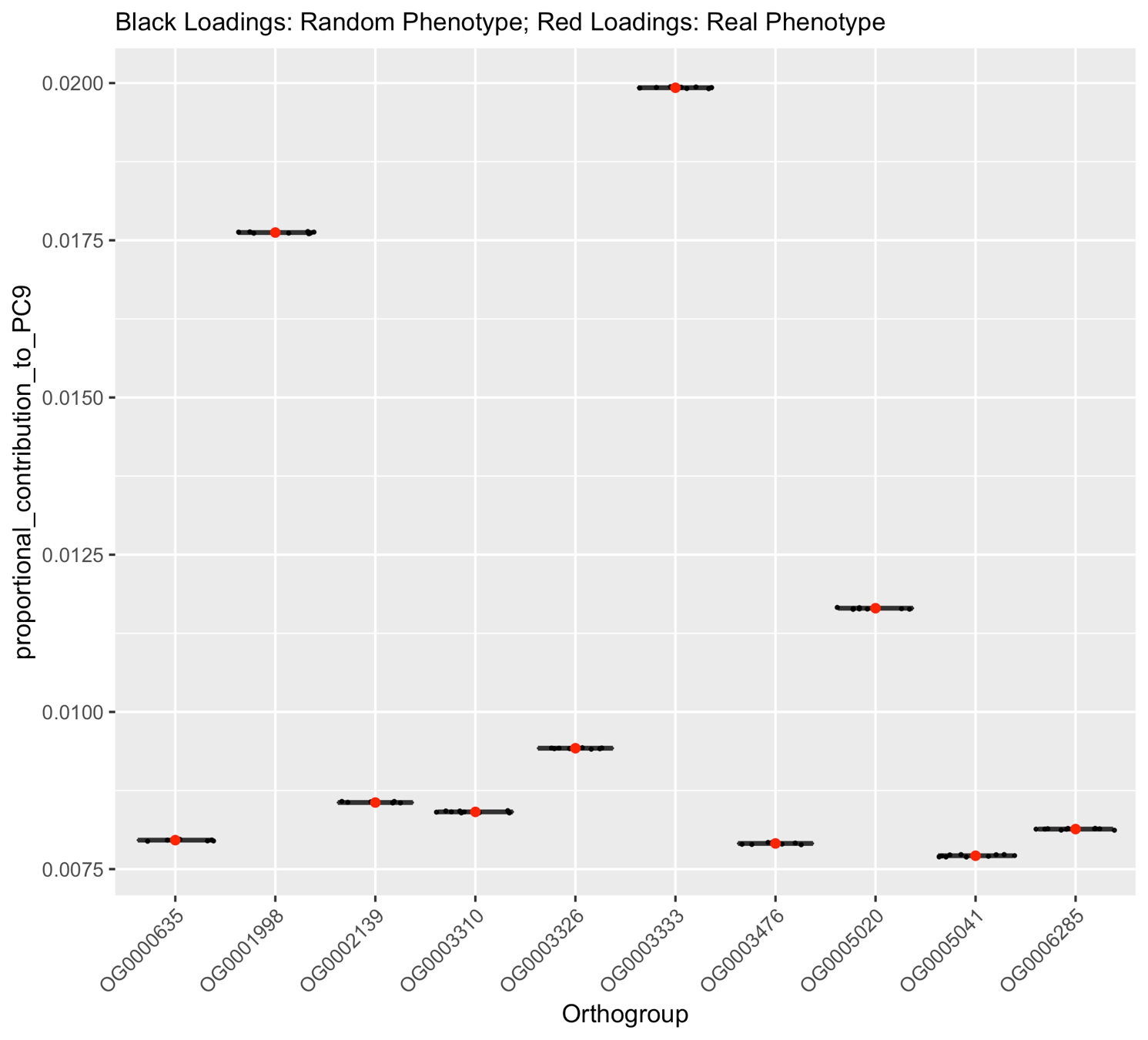


#### Supplementary Figure 23. PCA Randomization Tests

Samples from each focal species had their phenotype randomized prior to be tested by PCA over 10 iterations. For comparison purposes, we show in red the real data: specifically, the 10 orthogroups (x axis) which had the highest proportional contributions to PC9 loading (y axis) in the real data. We show in black the results from the randomization: there is no difference between real signal and randomisation.

**References**

[Afgan, Enis, Dannon Baker, Marius van den Beek, Daniel Blankenberg, Dave Bouvier, Martin Čech, John Chilton, et al. 2016. “The Galaxy Platform for Accessible, Reproducible and Collaborative Biomedical Analyses: 2016 Update.” *Nucleic Acids Research* 44 (W1): W3–10.](http://paperpile.com/b/zTstmx/Y8m3U)

[Alexa, Adrian, and Jorg Rahnenfuhrer. 2010. “topGO: Enrichment Analysis for Gene Ontology.” *R Package Version* 2 (0): 2010.](http://paperpile.com/b/zTstmx/0WGE3)

[Altschul, S. F., W. Gish, W. Miller, E. W. Myers, and D. J. Lipman. 1990. “Basic Local Alignment Search Tool.” *Journal of Molecular Biology* 215 (3): 403–10.](http://paperpile.com/b/zTstmx/5Sqsr)

[Ashburner, M., C. A. Ball, J. A. Blake, D. Botstein, H. Butler, J. M. Cherry, A. P. Davis, et al. 2000. “Gene Ontology: Tool for the Unification of Biology. The Gene Ontology Consortium.” *Nature Genetics* 25 (1): 25–29.](http://paperpile.com/b/zTstmx/TPgHG)

[Baccarella, Alyssa, Claire R. Williams, Jay Z. Parrish, and Charles C. Kim. 2018. “Empirical Assessment of the Impact of Sample Number and Read Depth on RNA-Seq Analysis Workflow Performance.” *BMC Bioinformatics* 19 (1): 423.](http://paperpile.com/b/zTstmx/d4XJ)

[Cortes, Corinna, and Vladimir Vapnik. 1995. “Support-Vector Networks.” *Machine Learning* 20 (3): 273–97.](http://paperpile.com/b/zTstmx/yX5Xx)

[Dhungel, Eliza, Yassin Mreyoud, Ho-Jin Gwak, Ahmad Rajeh, Mina Rho, and Tae-Hyuk Ahn. 2021. “MegaR: An Interactive R Package for Rapid Sample Classification and Phenotype Prediction Using Metagenome Profiles and Machine Learning.” *BMC Bioinformatics* 22 (1): 25.](http://paperpile.com/b/zTstmx/ubQyT)

[Durinck, Steffen, Yves Moreau, Arek Kasprzyk, Sean Davis, Bart De Moor, Alvis Brazma, and Wolfgang Huber. 2005. “BioMart and Bioconductor: A Powerful Link between Biological Databases and Microarray Data Analysis.” *Bioinformatics*  21 (16): 3439–40.](http://paperpile.com/b/zTstmx/61noU)

[Gene Ontology Consortium. 2021. “The Gene Ontology Resource: Enriching a GOld Mine.” *Nucleic Acids Research* 49 (D1): D325–34.](http://paperpile.com/b/zTstmx/KaVGr)

[Ghanat Bari, Mehrab, Choong Yong Ung, Cheng Zhang, Shizhen Zhu, and Hu Li. 2017. “Machine Learning-Assisted Network Inference Approach to Identify a New Class of Genes That Coordinate the Functionality of Cancer Networks.” *Scientific Reports* 7 (1): 6993.](http://paperpile.com/b/zTstmx/mCO9C)

[Johnston, Rachel A., Philippe Vullioud, Jack Thorley, Henry Kirveslahti, Leyao Shen, Sayan Mukherjee, Courtney M. Karner, Tim Clutton-Brock, and Jenny Tung. 2021. “Morphological and Genomic Shifts in Mole-Rat ‘Queens’ Increase Fecundity but Reduce Skeletal Integrity.” *eLife* 10 (April). https://doi.org/](http://paperpile.com/b/zTstmx/JAqTV)[10.7554/eLife.65760](http://dx.doi.org/10.7554/eLife.65760)[.](http://paperpile.com/b/zTstmx/JAqTV)

[Langfelder, Peter, and Steve Horvath. 2008. “WGCNA: An R Package for Weighted Correlation Network Analysis.” *BMC Bioinformatics* 9 (December): 559.](http://paperpile.com/b/zTstmx/I2J5)

[Langfelder, Peter, Rui Luo, Michael C. Oldham, and Steve Horvath. 2011. “Is My Network Module Preserved and Reproducible?” *PLoS Computational Biology* 7 (1): e1001057.](http://paperpile.com/b/zTstmx/uxn7)

[Larkin, Aoife, Steven J. Marygold, Giulia Antonazzo, Helen Attrill, Gilberto Dos Santos, Phani V. Garapati, Joshua L. Goodman, et al. 2021. “FlyBase: Updates to the Drosophila Melanogaster Knowledge Base.” *Nucleic Acids Research* 49 (D1): D899–907.](http://paperpile.com/b/zTstmx/Fmcam)

[Liang, Zhengzheng S., Heather R. Mattila, Sandra L. Rodriguez-Zas, Bruce R. Southey, Thomas D. Seeley, and Gene E. Robinson. 2014. “Comparative Brain Transcriptomic Analyses of Scouting across Distinct Behavioural and Ecological Contexts in Honeybees.” *Proceedings. Biological Sciences / The Royal Society* 281 (1797). https://doi.org/](http://paperpile.com/b/zTstmx/nPGWM)[10.1098/rspb.2014.1868](http://dx.doi.org/10.1098/rspb.2014.1868)[.](http://paperpile.com/b/zTstmx/nPGWM)

[Love, Michael, Simon Anders, and Wolfgang Huber. 2014. “Differential Analysis of Count Data--the DESeq2 Package.” *Genome Biology* 15 (550): 10–1186.](http://paperpile.com/b/zTstmx/skuKV)

[Lyne, Rachel, Richard Smith, Kim Rutherford, Matthew Wakeling, Andrew Varley, Francois Guillier, Hilde Janssens, et al. 2007. “FlyMine: An Integrated Database for Drosophila and Anopheles Genomics.” *Genome Biology* 8 (7): R129.](http://paperpile.com/b/zTstmx/YFcD6)

[Meyer, D., E. Dimitriadou, K. Hornik, A. Weingessel, F. Leisch, C. C. Chang, and C. C. Lin. 2015. “Misc Functions of the Department of Statistics, Probability Theory Group (formerly: E1071).” *Package e1071. TU Wien*.](http://paperpile.com/b/zTstmx/T4w1G)

[R Core Team. 2014. *R: A Language and Environment for Statistical Computing*.](http://paperpile.com/b/zTstmx/d8Ezg) [http://www.R-project.org/](http://www.r-project.org/)[.](http://paperpile.com/b/zTstmx/d8Ezg)

[Schaack, Dominik, Markus A. Weigand, and Florian Uhle. 2021. “Comparison of Machine-Learning Methodologies for Accurate Diagnosis of Sepsis Using Microarray Gene Expression Data.” *PloS One* 16 (5): e0251800.](http://paperpile.com/b/zTstmx/4x8g8)

[Simão, Felipe A., Robert M. Waterhouse, Panagiotis Ioannidis, Evgenia V. Kriventseva, and Evgeny M. Zdobnov. 2015. “BUSCO: Assessing Genome Assembly and Annotation Completeness with Single-Copy Orthologs.” *Bioinformatics*  31 (19): 3210–12.](http://paperpile.com/b/zTstmx/Puxh4)

[Supek, Fran, Matko Bošnjak, Nives Škunca, and Tomislav Šmuc. 2011. “REVIGO Summarizes and Visualizes Long Lists of Gene Ontology Terms.” *PloS One* 6 (7): e21800.](http://paperpile.com/b/zTstmx/HQe8O)

[Taylor, Benjamin A., Alessandro Cini, Christopher D. R. Wyatt, Max Reuter, and Seirian Sumner. 2021. “The Molecular Basis of Socially Mediated Phenotypic Plasticity in a Eusocial Paper Wasp.” *Nature Communications* 12 (1): 775.](http://paperpile.com/b/zTstmx/qyHLc)

[Vabalas, Andrius, Emma Gowen, Ellen Poliakoff, and Alexander J. Casson. 2019. “Machine Learning Algorithm Validation with a Limited Sample Size.” *PloS One* 14 (11): e0224365.](http://paperpile.com/b/zTstmx/bgnO2)

[Vojvodic, Svjetlana, Brian R. Johnson, Brock A. Harpur, Clement F. Kent, Amro Zayed, Kirk E. Anderson, and Timothy A. Linksvayer. 2015. “The Transcriptomic and Evolutionary Signature of Social Interactions Regulating Honey Bee Caste Development.” *Ecology and Evolution* 5 (21): 4795–4807.](http://paperpile.com/b/zTstmx/fgTyl)

[Wittwer, Bernadette, Abraham Hefetz, Tovit Simon, Li E. K. Murphy, Mark A. Elgar, Naomi E. Pierce, and Sarah D. Kocher. 2017. “Solitary Bees Reduce Investment in Communication Compared with Their Social Relatives.” *Proceedings of the National Academy of Sciences of the United States of America* 114 (25): 6569–74.](http://paperpile.com/b/zTstmx/mIMKg)

[Yuan, Fei, Lin Lu, and Quan Zou. 2020. “Analysis of Gene Expression Profiles of Lung Cancer Subtypes with Machine Learning Algorithms.” *Biochimica et Biophysica Acta, Molecular Basis of Disease* 1866 (8): 165822.](http://paperpile.com/b/zTstmx/rvmpS)

[Zararsız, Gökmen, Dincer Goksuluk, Selcuk Korkmaz, Vahap Eldem, Gozde Erturk Zararsiz, Izzet Parug Duru, and Ahmet Ozturk. 2017. “A Comprehensive Simulation Study on Classification of RNA-Seq Data.” *PLOS ONE*. https://doi.org/](http://paperpile.com/b/zTstmx/g13ur)[10.1371/journal.pone.0182507](http://dx.doi.org/10.1371/journal.pone.0182507)[.](http://paperpile.com/b/zTstmx/g13ur)
